# Mechanism of Gzma‐mediated GEF‐H1 activation in intestinal epithelial cells leading to intestinal barrier dysfunction in sepsis

**DOI:** 10.1002/ctm2.70651

**Published:** 2026-04-06

**Authors:** Zexing Lin, Haiyang Jiang, Chujun Ni, Liting Deng, Huan Yang, Runnan Wang, Peizhao Liu, Xuanheng Li, Yilong Yu, Weijie Li, Bo Liao, Juanhan Liu, Weizhen Li, Jiaxin Yang, Yue Chao, Haiqing Liu, Xiuwen Wu, Jianan Ren, Yun Zhao

**Affiliations:** ^1^ Department of General Surgery Nanjing BenQ Medical Center The Affiliated BenQ Hospital of Nanjing Medical University Nanjing China; ^2^ Department of Surgical Research Laboratory Nanjing BenQ Medical Center The Affiliated BenQ Hospital of Nanjing Medical University, The Clinical Translational Research Center for Surgical Infection and Immunity of Nanjing Medical University Nanjing China; ^3^ School of Medicine Southeast University Nanjing China; ^4^ Research Institute of General Surgery Affiliated Jinling Hospital, Medical School of Nanjing University Nanjing China; ^5^ Department of Critical Care Medicine The Affiliated Jiangning Hospital with Nanjing Medical University Nanjing China; ^6^ The Affiliated Hospital of Qingdao University Qingdao China

**Keywords:** Epothilone A, Epithelial integrity, Gzma (granzyme A), GEF‐H1, Intestinal barrier dysfunction, RhoA/ROCK signalling, Sepsis

## Abstract

**Background:**

Sepsis‐induced intestinal injury is a severe complication associated with dysfunction affecting multiple organ systems and a significantly elevated risk of death. Intestinal barrier dysfunction plays a central role, but the underlying molecular pathways remain incompletely understood. The present study sought to explore how the Gzma/GEF‐H1/RhoA signalling axis contributes to the disruption of the intestinal epithelial barrier in sepsis.

**Methods:**

Transcriptomic data, clinical samples, and a murine caecal ligation and puncture (CLP) model was used to assess Gzma expression and its correlation with disease severity. We investigated how Gzma—released by activated immune cells—affects epithelial structure and function using in vitro co‐culture assays. These experiments assessed key tight junction proteins (occludin, claudin‐1, ZO‐1, E‐cadherin), transepithelial electrical resistance (TEER), and paracellular permeability. GEF‐H1 knockout mice and the GEF‐H1 activator plinabulin were employed to evaluate the physiological roles of GEF‐H1. Mutagenesis revealed how Gzma activates GEF‐H1. High‐throughput screening identified a GEF‐H1 modulator, and its efficacy was validated in septic mice. Gzma expression was significantly elevated during sepsis and correlated with disease severity. Gzma secretion from immune cells impaired the epithelial barrier by downregulating tight junction proteins, increasing permeability, and reducing TEER. Gzma activates GEF‐H1 by dephosphorylating Ser886, triggering the RhoA/ROCK pathway and subsequent phosphorylation of MLC2, LIMK, and cofilin—driving cytoskeletal remodelling. GEF‐H1 knockout mice showed reduced intestinal injury, higher survival rates, and intact barrier function; conversely, GEF‐H1 activation worsened intestinal damage. High‐throughput screening identified Epothilone A as a potent GEF‐H1 modulator that restores intestinal barrier integrity and improves survival in murine sepsis by suppressing the GEF‐H1/librariesRhoA pathway.

**Conclusion:**

This research uncovers the Gzma/GEF‐H1/RhoA signalling axis as a pivotal contributor to intestinal barrier dysfunction during sepsis. GEF‐H1 represents a promising therapeutic target, and its inhibition by agents such as Epothilone A may offer a novel strategy for treating sepsis.

**Key points:**

- Gzma induces the dephosphorylation of Ser886 on GEF‐H1, activating the RhoA/ROCK pathway and disrupting the intestinal epithelial barrier.
- Knocking out GEF‐H1 can alleviate intestinal damage, protect multiple organs, and increase the survival rate of septic mice.
- Epothilone A inhibits the activation of GEF‐H1, thereby restoring the barrier function and reducing the mortality rate of sepsis.

## 1 INTRODUCTION

Sepsis is a major global public health emergency, with an estimated 48.9 million annual cases and 11 million deaths worldwide—accounting for 19.7% of all global deaths.^1^ Defined by the Sepsis‐3 Consensus as life‐threatening organ failure from an abnormal immune response to infection,^2^ sepsis often progresses to multiple organ dysfunction syndrome (MODS), with sepsis‐induced intestinal injury (SII) being a common and prognostically adverse complication.^3^ Occurring in 62%–85% of critically ill septic patients, advanced SII correlates strongly with immune dysfunction and elevated mortality,^4^, ^5^ highlighting the need to elucidate its molecular mechanisms for targeted therapeutic development.

Intestinal barrier dysfunction is a central pathological feature of sepsis, primarily manifested by the disruption of epithelial tight junctions, increased mucosal permeability, and subsequent bacterial or endotoxin translocation, which can further amplify systemic inflammatory responses and contribute to multi‐organ dysfunction.^6^ Growing research indicates that aberrant activation of Rho‐family GTPases is strongly associated with cytoskeletal remodelling and disruption of epithelial barrier function.^7^, ^8^ GEF‐H1, a guanine nucleotide exchange factor encoded by the ARHGEF2 gene, is a Rho‐specific GEF whose activity is modulated by microtubules. Functioning as a pivotal regulatory node, it bridges cytoskeletal reorganization with intracellular signal transduction pathways, making it crucial for preserving the structural and functional integrity of epithelial and endothelial barriers.^9^, ^10^ GEF‐H1 exhibits a modular organization with functionally specialized regions: the N‐terminal C1 domain anchors it to microtubules, the central DH domain serves as the catalytic core for RhoA activation, the PH domain stabilizes the DH domain and enables membrane recruitment, and the C‐terminal CC region and 14‐3‐3 interaction motif mediate regulatory interactions and activity control.^11^, ^12^, ^13^, ^14^

From a functional perspective, GEF‐H1 is subject to precise spatiotemporal control, largely dictated by the assembly and disassembly status of microtubules: it remains sequestered and inactive while associated with polymerized, stable microtubules, but becomes liberated and activated following microtubule disassembly, thereby initiating RhoA‐dependent signalling cascades.^15^ This distinctive regulatory mechanism allows GEF‐H1 to coordinate multiple cellular signals—such as mechanical strain, reactive oxygen species (ROS), and pathogen‐induced stress—that collectively trigger microtubule disassembly, thereby liberating GEF‐H1.^16^, ^17^, ^18^ In epithelial cells, GEF‐H1 modulates intercellular junctions and actin cytoskeletal dynamics by activating the RhoA/ROCK pathway, thereby regulating barrier integrity.^9^, ^10^ This unique regulatory pathway allows GEF‐H1 to coordinate multiple cellular signals—such as mechanical strain and ROS, and stress elicited by pathogens—all of which converge to induce microtubule depolymerization and subsequently release GEF‐H1.^19^, ^20^, ^21^ Importantly, under inflammatory conditions, TNF‐α triggers p38MAPK activation, which in turn phosphorylates GEF‐H1 at Ser885. This post‐translational modification drives the redistribution of GEF‐H1 from microtubules to the plasma membrane, thereby stimulating RhoA and Rac1 signalling—ultimately leading to tight junction disassembly and enhanced vascular leakage.^9^ However, the exact role of GEF‐H1 in sepsis‐induced intestinal epithelial barrier dysfunction, particularly its interplay with sepsis‐induced inflammatory pathways, remains incompletely characterized, representing a key gap in knowledge regarding immune–epithelial crosstalk in sepsis.

It should be emphasized that various pathogens can exacerbate tissue damage in sepsis by hijacking host cell signalling pathways.^22^ For example, Pseudomonas aeruginosa employs the type III secretion apparatus to deliver virulence‐associated effector molecules, such as ExoT and ExoS, into host cells, thereby disrupting the cytoskeletal structure and compromising the integrity of the epithelial barrier.^23^, ^24^ Concurrently, granzyme A (Gzma)—a serine protease released by cytotoxic T cells and natural killer (NK) cells—has been newly identified as a mediator of inflammation and tissue injury via non‐apoptotic pathways, expanding beyond its well‐established function in inducing apoptosis.^25^, ^26^ Gzma can cleave multiple cellular substrates to modulate signalling cascades, and its involvement in cytoskeletal reorganization and barrier dysfunction has been suggested in inflammatory models.^25^ However, whether Gzma influences intestinal epithelial barrier function via the regulation of GEF‐H1 activity—such as direct cleavage, modification of its regulatory domains, or indirect modulation of its microtubule binding and activation—remains to be elucidated.

This research explores how the Gzma/GEF‐H1/RhoA signalling pathway contributes to intestinal barrier impairment in sepsis. We hypothesize that, within the septic microenvironment, Gzma may activate GEF‐H1 in intestinal epithelial cells through either direct interaction or indirect regulatory mechanisms, thereby activating the RhoA/ROCK signalling pathway. This activation leads to an enhanced phosphorylated form of the myosin light chain (p‐MLC), disorganization of critical tight junction components—such as occludin and claudin‐1—and ultimately compromises the structural and functional competence of the epithelial barrier. Given the critical role of GEF‐H1 in bridging extracellular stimuli and cytoskeletal dynamics, elucidating how Gzma modulates its activity in sepsis could yield both a fresh mechanistic insight into sepsis‐induced intestinal damage and a conceptual basis for designing therapeutics that specifically target the GEF‐H1 pathway—such as peptide inhibitors targeting its catalytic domain or microtubule‐binding region^27^—thereby holding substantial potential for translational medicine.

## 2 RESULTS

### 2.1 Elevated expression of Gzma in sepsis and its mediation of intestinal epithelial barrier dysfunction

**FIGURE 1:**
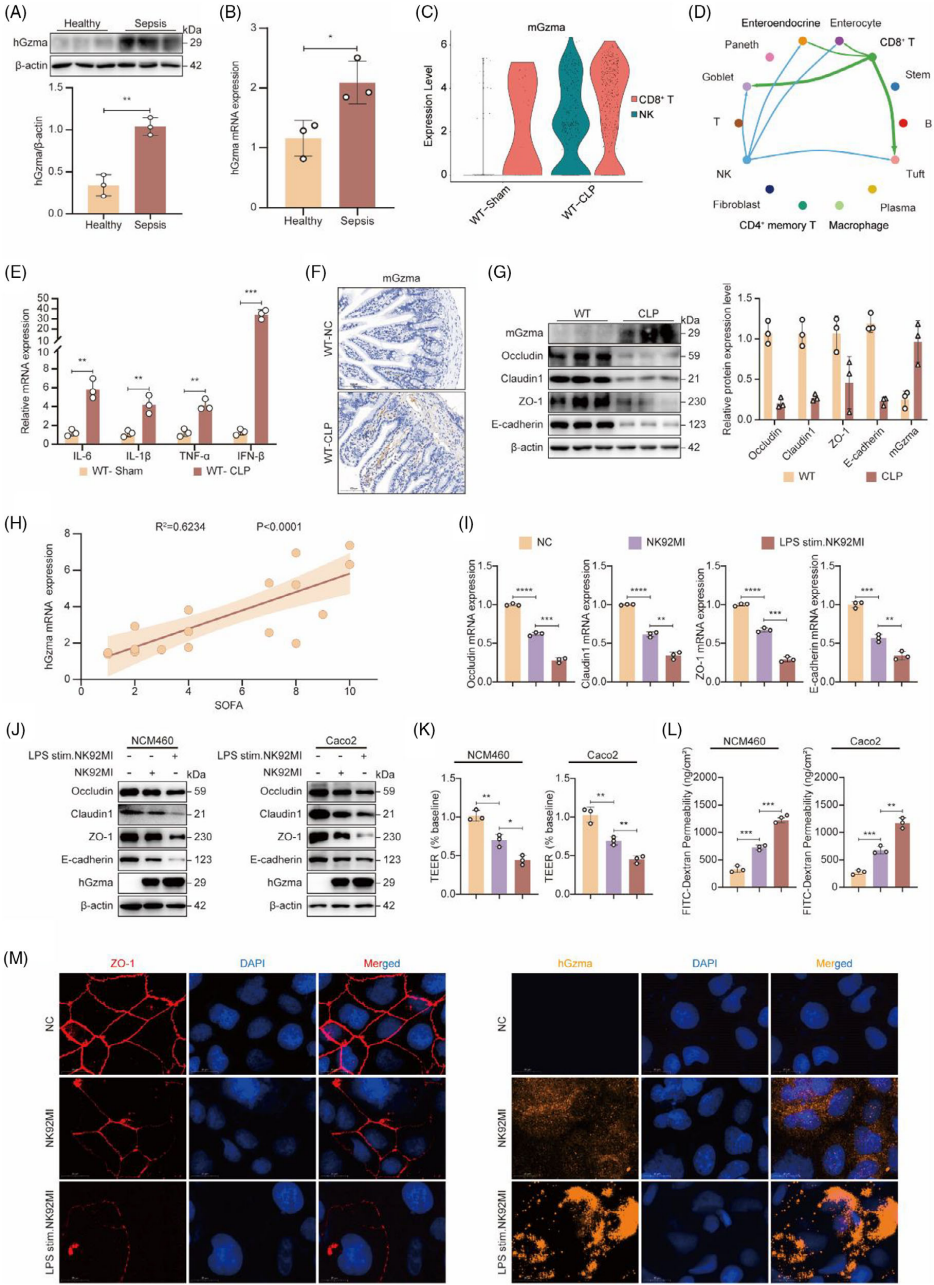
Elevated expression of Gzma in sepsis and its mediation of intestinal epithelial barrier dysfunction. (A) Western blot analysis of Gzma and β‐actin in PBMCs from healthy controls and septic patients. Data: mean ± SEM; *n* = 3 per group. Student's *t*‐test, **p < .01. (B) RT‐qPCR analysis of Gzma mRNA in PBMCs from both groups. Data: mean ± SEM; *n* = 3. *p < .05 by Student's *t*‐test. (C) Violin plots showing Gzma expression in NK and CD8^+^ T cells from WT‐Sham and WT‐CLP mice, based on scRNA‐seq of intestinal tissues. (D) Cell–cell interaction network in intestinal tissue under sepsis. Line thickness indicates interaction strength. (E) RT‐qPCR analysis of pro‐inflammatory cytokines (IL‐6, IL‐1β, TNF‐α, IFN‐β) in intestinal tissues from WT‐Sham and WT‐CLP mice. Data: mean ± SEM; *n* = 3. One‐way ANOVA with Tukey's test. (F) IHC staining showing Gzma localization in intestinal tissues. Scale bar: 100 µm. (G) Western blot analysis of Gzma, tight junction proteins (Claudin1, ZO‐1, Occludin), and E‐cadherin in intestinal tissues. Right panel: densitometry normalized to β‐actin. Data: mean ± SEM; *n* = 3. ***p < .001, **p < .01, *p < .05 by Student's *t*‐test. (H) Correlation between Gzma mRNA in septic patient PBMCs and SOFA scores: Pearson *r* = .6234, *p* < .0001. (I) RT‐qPCR analysis of tight junction proteins (Occludin, Claudin1, ZO‐1) and E‐cadherin in NCM460 cells co‐cultured with NC. Data: mean ± SEM; *n* = 3. ****p < .0001, ***p < .001, **p < .01 by one‐way ANOVA. (J) Protein levels of intestinal epithelial barrier‐related molecules (Occludin, Claudin1, ZO‐1, E‐cadherin) in NCM460/Caco2 cells decreased after co‐culture; Gzma secretion by LPS‐stimulated NK92MI cells increased. (K) TEER of NCM460/Caco2 cells was significantly reduced in the co‐culture system. Data: mean ± SEM; *n* = 3. **p < .01, *p < .05 by one‐way ANOVA. (L) FITC‐dextran permeability of NCM460/Caco2 cells was markedly elevated in the co‐culture system. Data: mean ± SEM; *n* = 3. ***p < .001, **p < .01 by one‐way ANOVA. (M) Immunofluorescence showed downregulated ZO‐1 in Caco2 cells (left, red) and increased Gzma secretion by LPS‐stimulated NK92MI cells (right, orange), confirming impaired barrier integrity. Scale bar: 20 µm.

To explore the role of Gzma in maintaining intestinal barrier integrity dysfunction during sepsis, we initially examined publicly available transcriptomic data from mononuclear cells isolated from the peripheral blood of 393 human subjects, as reported by Arjun Baghela and colleagues.^28^ Our analysis revealed notable differences between healthy controls and the study cohort; the blood of sepsis patients exhibited a marked upregulation of Gzma at the transcriptional level (Figure S1A). Given the difficulty in obtaining human intestinal tissue samples, we further examined the expression of Gzma in PBMCs from sepsis patients. The findings revealed a significant upregulation of Gzma at both the protein and transcript levels compared with healthy controls (Figure 1A,B). Furthermore, based on the single‐cell transcriptomic analysis of CLP‐induced mouse intestinal tissue from our group's previous work, our analysis revealed a marked increase in Gzma expression inside NK cells and cytotoxic T lymphocytes isolated from CLP mice relative to those from wild‐type (WT) controls. Notably, these immune cell populations exhibited extensive interactions with various intestinal epithelial cell types (Figure 1C,D).

Next, we successfully generated a murine sepsis model using caecal ligation and puncture (CLP). HE‐dye‐based tissue visualization combined with microscopic pathological analysis showed pronounced structural injury in the intestinal, hepatic, and pulmonary tissues of CLP‐challenged mice (Figure S1B). Moreover, the concentrations of critical inflammatory mediators—including IL‐6, IL‐1β, TNF‐α, and IFN‐β—were markedly increased (Figure 1E), confirming the successful establishment of the model. Immunohistochemical staining demonstrated increased expression of Gzma in the gut tissue of mice subjected to CLP (Figure 1F). Furthermore, both the expression levels of Gzma at both the protein and mRNA were significantly increased in the intestinal tissue of mice subjected to CLP, whereas the abundance of critical proteins linked to tight junctions—namely, occludin, claudin‐1, and ZO‐1 and the cell adhesion protein E‐cadherin were significantly reduced at both the protein and transcriptional levels (Figure 1G; Figure S1C). Immunofluorescence staining further validated the decreased levels of ZO‐1 and E‐cadherin, indicating structural injury to the intestinal epithelial barrier (Figure S1D). Collectively, these findings suggest that elevated levels of Gzma are associated with the observed effects and that these effects are linked to damage of the intestinal epithelial barrier in sepsis.

To further investigate the possible link between Gzma and damage to the intestinal barrier, we analyzed clinical samples from sepsis patients and found that the mRNA levels of Gzma, IL‐1β, and TNF‐α in peripheral blood exhibited a significant positive association with the SOFA score (Figure 1H; Figure S1E), indicating that Gzma could potentially contribute to the worsening of intestinal barrier dysfunction. To evaluate its functional role, we co‐cultured LPS‐pretreated NK92MI cells, which mimic the activated state of NK cells during sepsis, with three intestinal epithelial cell lines (NCM460, HT‐29, and Caco2), respectively. In intestinal epithelial cells, the expression—at both the protein and transcript levels—of critical junctional proteins, for instance Occludin, Claudin‐1, ZO‐1, and E‐cadherin, was substantially downregulated. Concurrently, LPS‐activated NK92MI cells exhibited a pronounced elevation in Gzma release (Figure 1I,J; Figure S1F). Moreover, the co‐culture model demonstrated a marked reduction in transepithelial electrical resistance (TEER) across intestinal epithelial monolayers, accompanied by elevated paracellular flux of FITC‐labelled dextran (Figure 1K,L; Figure S1G,H). Immunofluorescence staining further confirmed the downregulation of ZO‐1 and E‐cadherin expression, indicating compromised barrier integrity (Figure 1M; Figure S1I).

To directly assess the effect of Gzma on the intestinal epithelial barrier, this study followed the method reported by Shao et al.,^29^ and used electroporation technology to transfect Gzma into three different intestinal epithelial cell lines. The results showed that the upregulation of Gzma expression could significantly downregulate the expression of tight junction‐related proteins Occludin, Claudin‐1, ZO‐1, and E‐cadherin at both the protein and transcriptional levels (Figure S1J,K). The above results suggest that in the context of sepsis, the increased expression of Gzma can directly damage the structural and functional integrity of the intestinal epithelial barrier.

### 2.2 Activation of the GEF‐H1/RhoA signalling axis by Gzma under sepsis conditions

**FIGURE 2:**
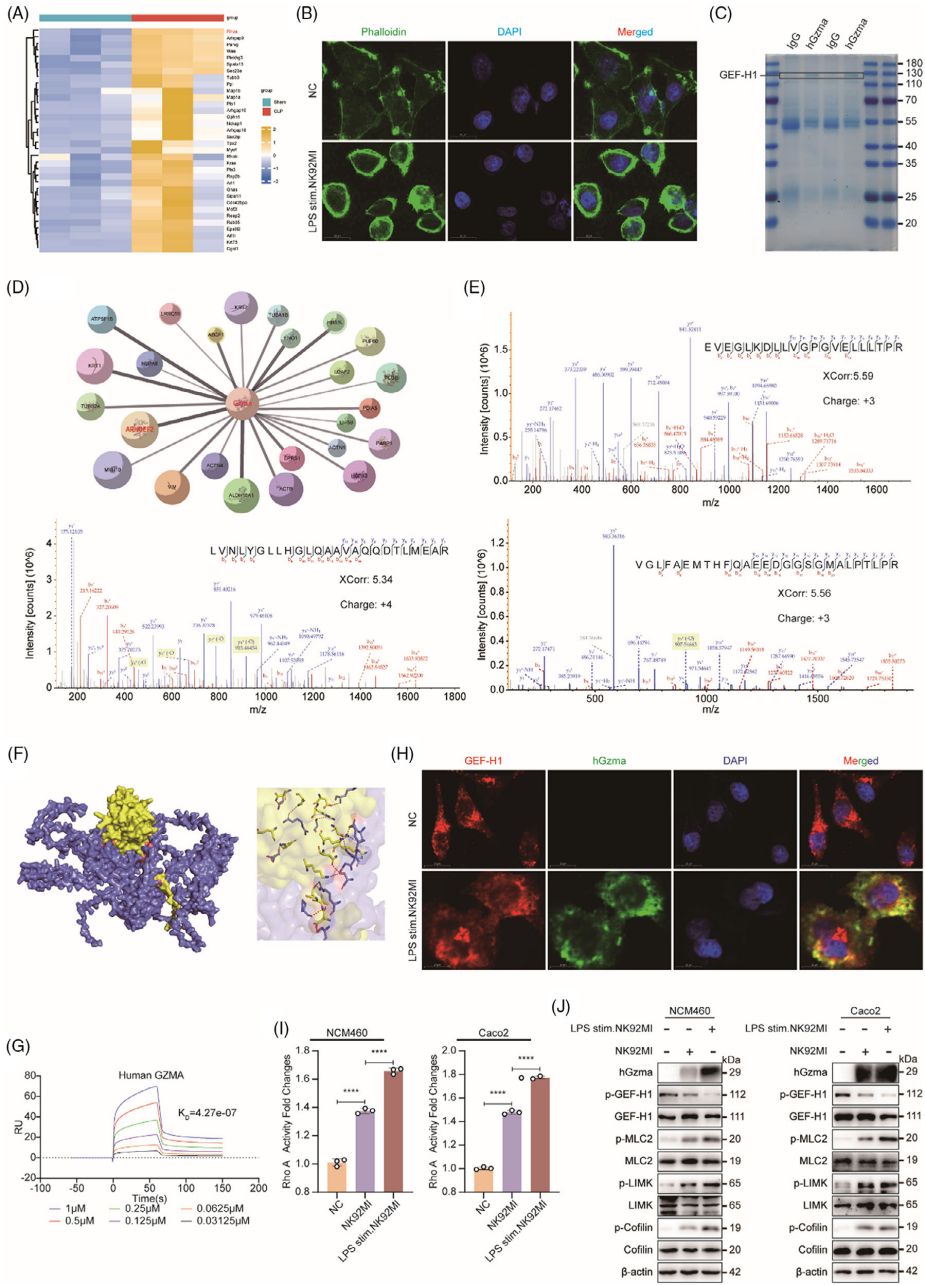
Activation of the GEF‐H1/RhoA signalling axis by Gzma under sepsis conditions. (A) Heatmap of transcriptome sequencing analysis showing differential expression profiles of small GTPases and cytoskeleton‐related genes in the intestinal tissues of WT‐Sham and the CLP sepsis model group (rows: genes; columns: experimental groups; colour scale: relative expression level). (B) Immunofluorescence staining of phalloidin (green, labelling F‐actin) in human colonic epithelial NCM460 cells after co‐culture with LPS‐pretreated NK92MI cells (DAPI staining for nuclei, blue; top row: control group; bottom row: co‐culture group; scale bar = 20 µm). (C) Coomassie blue‐stained SDS‐PAGE gel following Gzma immunoprecipitation (IP); the arrow indicates the GEF‐H1 band. (D) Interaction network between Gzma and candidate binding proteins predicted by the STRING database (Nodes: proteins). (E) Mass spectrum (MS/MS) of high‐affinity peptides binding Gzma to GEF‐H1, showing peptide sequences (EVEGLKDLLVGPGVELLLTPR, LVNLYGLLHGLQAAVAQQDTLMEAR, VGLFAEMTHFQAEEDGGSGMALPTLPR), Xcorr (cross‐correlation coefficient), and charge state. (F) Left: The three‐dimensional structure of the molecular docking of Gzma (yellow) and GEF‐H1 (blue) simulated by the GRAMM software; Right: A close‐up view highlighting the key interacting residues at the interface. (G) SPR analysis of GEF‐H1 binding to Gzma. Sensorgrams show concentration‐dependent binding of Gzma (.125–1 µM). The measured *K*_D_ is 4.27⨯10^−7^ M, indicating a medium‐high affinity interaction. (H) Immunofluorescence co‐localization analysis of GEF‐H1 (red) and Gzma (green) in NCM460 cells after co‐culture with LPS‐pretreated NK cells (DAPI staining for nuclei, blue; top row: control group; bottom row: co‐culture group; scale bar = 20 µm). (I) RhoA G‐LISA activity assay in NCM460 (left) and Caco2 (right) cells after co‐culture with LPS‐stimulated NK cells (mean ± SEM, *n* = 3; ****p < .0001, one‐way ANOVA). (J) Western blot analysis of GEF‐H1, p‐GEF‐H1 (Ser886), MLC2, p‐MLC2 (Thr18/Ser19), LIMK, p‐LIMK (Thr508), Cofilin, p‐Cofilin (Ser3), and β‐actin (used as a loading control) was performed in NCM460 and Caco2 cells following co‐culture with LPS‐stimulated NK cells.

To gain deeper insights into how Gzma mediates damage to the intestinal epithelial barrier during sepsis at the molecular level, we established a CLP‐induced murine sepsis model and conducted transcriptomic sequencing analysis on intestinal tissues. The findings revealed a marked increase in the levels of multiple small GTP‐binding proteins and associated genes associated with cytoskeletal regulation in the intestinal tissues of mice subjected to CLP, relative to WT controls. Notably, the change in RhoA expression was particularly pronounced (Figure 2A), suggesting that RhoA may play a pivotal role in involved in the disruption of intestinal barrier integrity during sepsis.

To simulate immune cell–epithelial cell interactions within the septic microenvironment in vitro, we co‐cultured LPS‐pretreated NK92MI cells with human colonic epithelial NCM460 cells and performed transcriptomic sequencing analysis. GO enrichment analysis revealed notable alterations in biological pathways such as “microtubule morphogenesis,” “regulation of cell dynamics,” and “maintenance of epithelial structure” in the co‐culture group (Figure S2A), along with markedly upregulated expression levels of multiple cytoskeleton‐related genes (Figure S2B). Moreover, immunofluorescence staining using phalloidin and β‐tubulin demonstrated that NCM460 cells in the co‐culture group transitioned from polygonal or spindle‐like morphologies to rounded shapes (Figure 2B; Figure S2C), suggesting substantial cytoskeletal remodelling. Collectively, these findings indicate that Gzma may contribute to epithelial barrier dysfunction under septic conditions by modulating cytoskeletal dynamics.

Considering that GEF‐H1 functions as a critical GEF responsible for activating RhoA, which exerts its function through facilitating the substitution of GDP with GTP, thereby initiating downstream signalling pathways, we hypothesized that Gzma might regulate RhoA activation via direct binding to GEF‐H1. To validate this hypothesis, we first examined the functional crosstalk between Gzma and GEF‐H1 using mass spectrometry (Figure 2C). The results revealed a strong binding affinity between the two proteins, with three high‐affinity binding peptides identified: EVEGLKDLLVGPGVELLLTPR, LVNLYGLLHGLQAAVAQQDTLMEAR, and VGLFAEMTHFQAEEDGGSGMALPTLPR (Figure 2D,E). The protein–protein molecular docking simulation conducted using the GRAMM software further indicates that the binding free energy between Gzma and GEF‐H1 is –11.6 kcal/mol. Generally, a binding free energy lower than –4 kcal/mol is considered biologically feasible, and the more negative the value, the more stable the protein interaction. The current results strongly support the existence of a specific and stable interaction between Gzma and GEF‐H1 (Figure 2F). Additionally, the surface plasmon resonance (SPR) experiment measurement shows that the affinity constant KD of GEF‐H1 and Gzma is 4.27 × 10^−^^7^ M, which falls within the medium‐high affinity range (Figure 2G). Immunofluorescence co‐localization analysis further confirmed significant co‐localization of Gzma and GEF‐H1 in intestinal epithelial cells under co‐culture conditions. Notably, an enhanced Gzma fluorescence signal was accompanied by marked morphological changes, as cells transitioned from a spindle‐shaped to a rounded morphology (Figure 2H), further indicating their functional interaction and potential regulatory role in cytoskeletal remodelling.

A substantial body of research has confirmed that the critical involvement of the RhoA/ROCK signalling cascade modulates downstream effectors such as LIMK/Cofilin and MLC2 through phosphorylation, thereby promoting actin cytoskeletal remodelling, enhancing myosin contractility, and ultimately compromising epithelial barrier function.^19^, ^20^, ^21^ To further explore the downstream mechanisms of the Gzma/GEF‐H1 interaction in sepsis‐induced intestinal epithelial barrier injury, we co‐cultured LPS‐pretreated NK cells with three human intestinal epithelial cell lines (NCM460, HT‐29, and Caco2). RhoA G‐LISA activity assays and Western blot analyses revealed significantly elevated RhoA activity, along with GEF‐H1 dephosphorylation and activation, along with elevated phosphorylation of myosin light chain 2 (MLC2), LIM kinase (LIMK), and cofilin in the co‐cultured cells (Figure 2I,J; Figure S2D,E), suggesting the involvement of two distinct signalling cascades: RhoA/ROCK/LIMK/Cofilin and RhoA/ROCK/MLC2. To confirm the direct involvement of Gzma, we performed electroporation of Gzma plasmids into the three intestinal epithelial cell lines. Similarly, increased RhoA activity, GEF‐H1 dephosphorylation and activation, and consistent upregulation of MLC2, LIMK, and Cofilin phosphorylation were observed (Figure S2F,G). Collectively, these findings indicate that Gzma directly interacts with GEF‐H1, activates RhoA and its downstream signalling cascades, and thereby induces cytoskeletal remodelling.

### 2.3 Protective effect of GEF‐H1 inhibition on sepsis‐associated epithelial barrier damage

**FIGURE 3:**
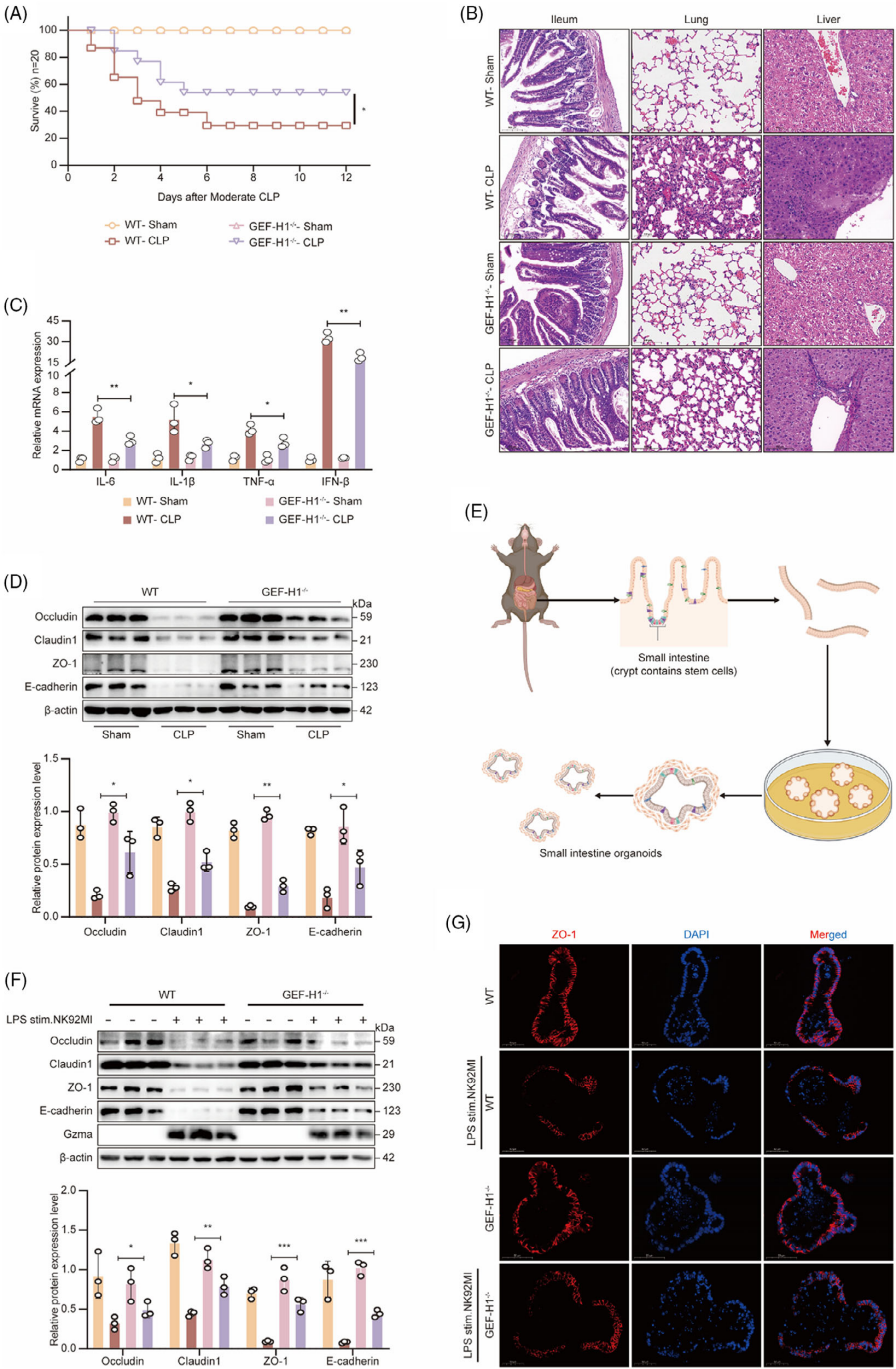
Protective effect of GEF‐H1 inhibition on sepsis‐associated epithelial barrier damage. (A) Survival curves of WT and GEF‐H1^−/−^ mice after CLP‐induced sepsis (*n* = 20 per group; *p < .05, Log‐rank test). (B) Histological analysis via H&E staining of ileum, lung, and liver tissues from WT and GEF‐H1^−/−^ mice following sham or CLP surgery (scale bar = 100 µm). (C) RT‐qPCR assessment of inflammatory cytokine expression (IL‐6, IL‐1β, TNF‐α, IFN‐β) in ileal tissues from WT and GEF‐H1^−/−^ mice after sham or CLP treatment (mean ± SEM, *n* = 6 per group; *p < .05, **p < .01, two‐way ANOVA). (D) Western blot detection (upper panels) and corresponding grayscale quantification (lower panels) of tight junction proteins (Occludin, Claudin1, ZO‐1) and the adherens junction protein E‐cadherin in ileal tissues of WT and GEF‐H1^−/−^ mice after sham or CLP treatment (mean ± SEM, *n* = 3 per group; *p < .05, **p < .01, two‐way ANOVA). (E) Schematic illustration of the intestinal organoid isolation and culture protocol. (F) Western blot analysis (upper panels) and grayscale quantification (lower panels) of tight junction proteins (Occludin, Claudin1, ZO‐1) and E‐cadherin in WT and GEF‐H1^−/−^ intestinal organoids co‐cultured with LPS‐stimulated NK92MI cells (mean ± SEM, *n* = 3 per group; *p < .05, **p < .01, ***p < .001, two‐way ANOVA). (G) Immunofluorescence staining showing ZO‐1 localization in WT and GEF‐H1^−/−^ intestinal organoids co‐cultured with LPS‐stimulated NK92MI cells (blue: DAPI; red: ZO‐1; scale bar = 20 µm).

To explore whether Gzma contributes to intestinal barrier dysfunction during sepsis by triggering the RhoA/ROCK signalling cascade via GEF‐H1, we established a murine experimental model with genetic deletion of GEF‐H1 (GEF‐H1^−/−^) (Figure S3A) and induced sepsis using the CLP model. Survival analysis demonstrated that GEF‐H1 deficiency markedly enhanced the survival probability of septic mice (*p* < .05), underscoring the critical involvement of GEF‐H1 in sepsis development (Figure 3A). Histopathological analysis further revealed that intestinal tissue damage was markedly reduced in GEF‐H1^−/−^ mice versus wild‐type septic mice, as evidenced by better‐preserved intestinal villi, decreased epithelial disruption, and significantly less inflammatory cell infiltration. Additionally, in both lung and liver tissues, GEF‐H1 knockout substantially ameliorated sepsis‐induced pathological changes, including inflammatory infiltration, tissue oedema, hepatocellular necrosis, and vacuolar degeneration (Figure 3B), suggesting that the ablation of GEF‐H1 may confer protective effects across multiple organs.

At the molecular level, qPCR revealed a marked downregulation of gene expression profiling for key cytokines that promote inflammatory responses—including TNF‐α and IL‐6—in the intestinal tissues of GEF‐H1 knockout mice, indicating a suppression of both systemic and local inflammatory responses (Figure 3C). Importantly, both Western blot and qPCR analyses consistently showed that GEF‐H1 deficiency markedly enhanced the abundance of critical tight junction proteins—including occludin, claudin‐1, and ZO‐1—along with the cell adhesion molecule E‐cadherin (Figure 3D; Figure S3B), thereby providing molecular evidence supporting the restoration of intestinal barrier integrity when GEF‐H1 is absent.

To further confirm that GEF‐H1 plays a direct functional role in Gzma‐induced intestinal epithelial barrier injury and to circumvent the limitations associated with precisely modulating immune cell–epithelial cell interactions in vivo, we developed an in vitro co‐culture model comprising intestinal organoids and NK92MI cells (Figure 3E). In this system, LPS‐pretreated NK92MI cells induced significant barrier disruption in the organoids. However, in GEF‐H1 knockout organoids, the expression levels of tight junction and adhesion molecules remained notably higher (Figure 3F). Immunofluorescence staining further demonstrated that the continuity and distribution of ZO‐1 were largely preserved under GEF‐H1‐deficient conditions (Figure 3G), thereby confirming that GEF‐H1 functions as a key downstream signalling mediator through which Gzma compromises intestinal barrier integrity.

To conclude, this study underscores the essential function of GEF‐H1 in intestinal barrier injury associated with sepsis and indicates that inhibiting GEF‐H1 could represent a viable therapeutic strategy for mitigating multi‐organ dysfunction in sepsis.

### 2.4 Dephosphorylation‐mediated activation of GEF‐H1 exacerbates sepsis‐induced disruption of epithelial barrier integrity

**FIGURE 4:**
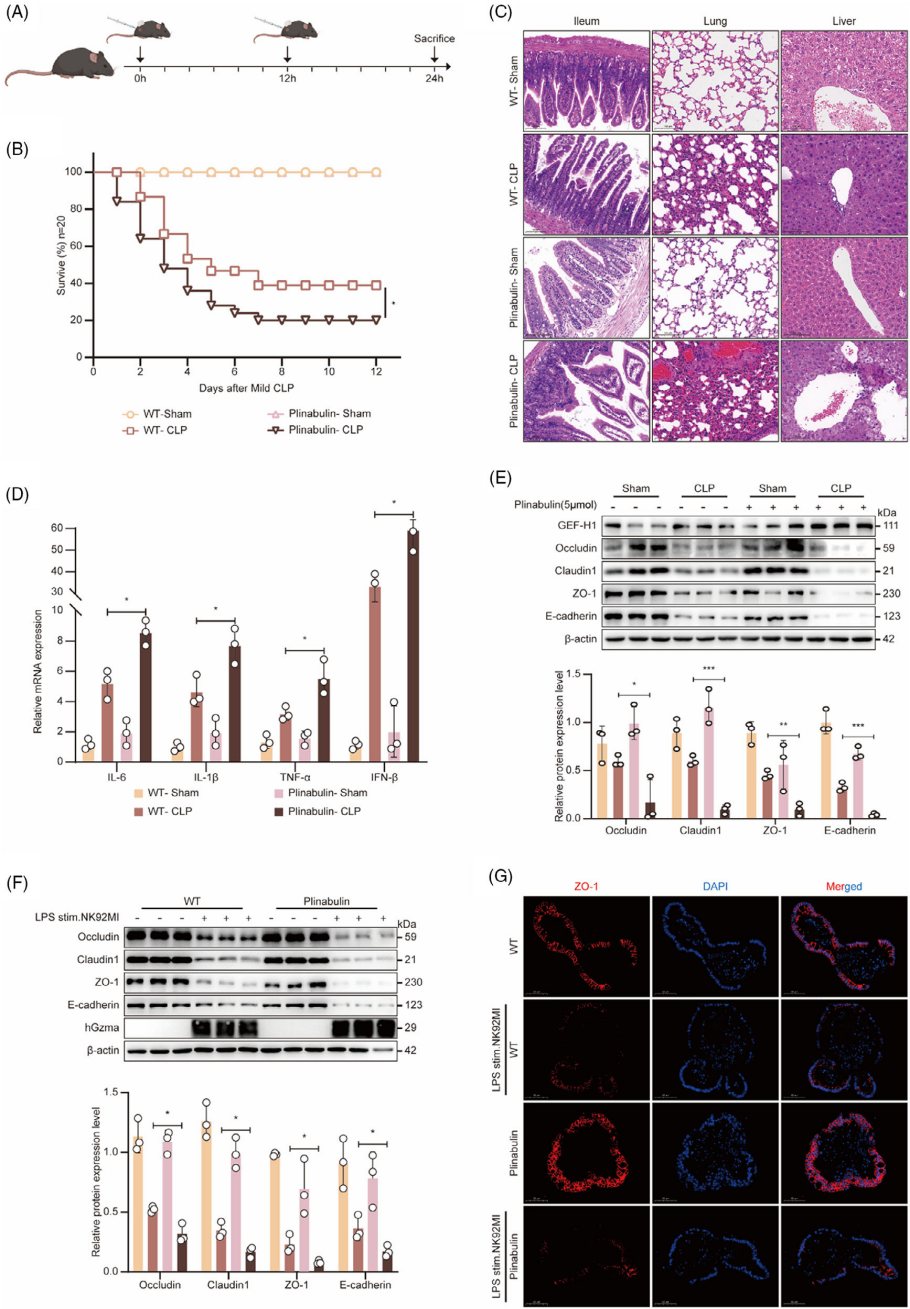
Dephosphorylation‐mediated activation of GEF‐H1 exacerbates sepsis‐induced disruption of epithelial barrier integrity. (A) Schematic diagram illustrating the experimental timeline in mice, including the time points for CLP modelling and Plinabulin intervention in WT mice (0 h: model establishment; 0 h and 12 h: intervention; 24 h: sample collection). (B) Survival curves of WT mice following sham operation (Sham), CLP, or CLP combined with Plinabulin treatment (*n* = 20 per group; *p < .05, Log‐rank test). (C) Histopathological analysis via HE staining of ileum, lung, and liver tissues from WT mice after Sham, CLP, or CLP + Plinabulin treatment (scale bar = 100 µm). (D) RT‐qPCR analysis of pro‐inflammatory cytokines (IL‐6, IL‐1β, TNF‐α, IFN‐β) in ileal tissues of WT mice after Sham, CLP, or CLP + Plinabulin treatment (mean ± SEM, *n* = 3 per group; *p < .05, two‐way ANOVA). (E) Western blot analysis (top) and corresponding grayscale quantification (bottom) of GEF‐H1, tight junction proteins (Occludin, Claudin1, ZO‐1), and the adhesion molecule E‐cadherin in ileal tissues of WT mice after Sham, CLP, or CLP + Plinabulin treatment (mean ± SEM, *n* = 3 per group; *p < .05, **p < .01, ***p < .001, two‐way ANOVA). (F) Western blot analysis (top) and grayscale quantification (bottom) of tight junction proteins (Occludin, Claudin1, ZO‐1) and E‐cadherin in intestinal organoids treated with vehicle (DMSO, PEG300, and Tween‐80) or Plinabulin (mean ± SEM, *n* = 3 per group; *p < .05, two‐way ANOVA). (G) Immunofluorescence staining of ZO‐1 in small intestinal organoids treated with vehicle (DMSO, PEG300, and Tween‐80) or Plinabulin (blue: DAPI for nuclei; red: ZO‐1; scale bar = 20 µm).

To further validate Gzma's involvement in compromising the integrity of the intestinal barrier by stimulating the GEF‐H1/RhoA/ROCK signalling pathway, we adopted a pharmacological gain‐of‐function strategy in a CLP‐induced murine sepsis model. Specifically, Plinabulin—a known GEF‐H1 activator—was administered intraperitoneally at a quantity of 15 mg/kg at the time of CLP surgery (Figure 4A). The findings revealed that Plinabulin administration led to a marked reduction in survival rates relative to the control group (Figure 4B), indicating that overactivation of GEF‐H1 may worsen the prognosis of sepsis. Subsequently, we performed a systematic histological evaluation of major organs.

Histopathological analysis revealed more severe multi‐organ damage in the Plinabulin‐treated group, including significant disruption of the intestinal mucosal structure, disordered arrangement of intestinal villi, extensive fragmentation and shortening; aggravated pulmonary oedema with substantial inflammatory cell infiltration; and more pronounced vacuolar degeneration and focal necrosis of hepatocytes in the liver (Figure 4C). These findings collectively demonstrate that GEF‐H1 activation synergizes with Gzma to exacerbate pathological organ damage during sepsis.

At the molecular level, qPCR assays demonstrated that Plinabulin administration markedly enhanced the expression of key pro‐inflammatory cytokines—namely TNF‐α, IL‐6, IFN‐β, and IL‐1β—in the intestinal tissue. (Figure 4D), suggesting an enhancement of inflammatory responses. More importantly, both Western blot and qPCR results consistently demonstrated that Plinabulin treatment markedly downregulated the levels of key intercellular junctional proteins—such as Occludin, Claudin‐1, and ZO‐1, as well as E‐cadherin (Figure 4E; Figure S3C), indicating a substantial disruption of intestinal epithelial barrier integrity at the molecular level.

To further validate this mechanism in an in vitro model, we performed experiments using intestinal organoids and observed that Plinabulin administration markedly decreased the expression of the specified tight junction and adherens junction proteins and their corresponding mRNAs, consistent with the observed in vivo effects (Figure 4F). Furthermore, immunofluorescence staining demonstrated that ZO‐1, which typically displays a continuous and well‐organized membranous localization, became fragmented and irregularly distributed following Plinabulin treatment, providing morphological evidence of epithelial barrier disruption (Figure 4G).

In summary, these experimental results consistently demonstrate at multiple levels that the GEF‐H1 agonist Plinabulin exacerbates Gzma‐induced intestinal barrier dysfunction by enhancing RhoA/ROCK pathway activity, thereby providing solid experimental evidence for the pivotal involvement of the Gzma‐mediated signalling cascade involving GEH‐H1 and the downstream RhoA/ROCK axis in intestinal damage during sepsis.

### 2.5 In vitro validation of GEF‐H1's essential role in Gzma‐mediated intestinal barrier injury during sepsis

**FIGURE 5:**
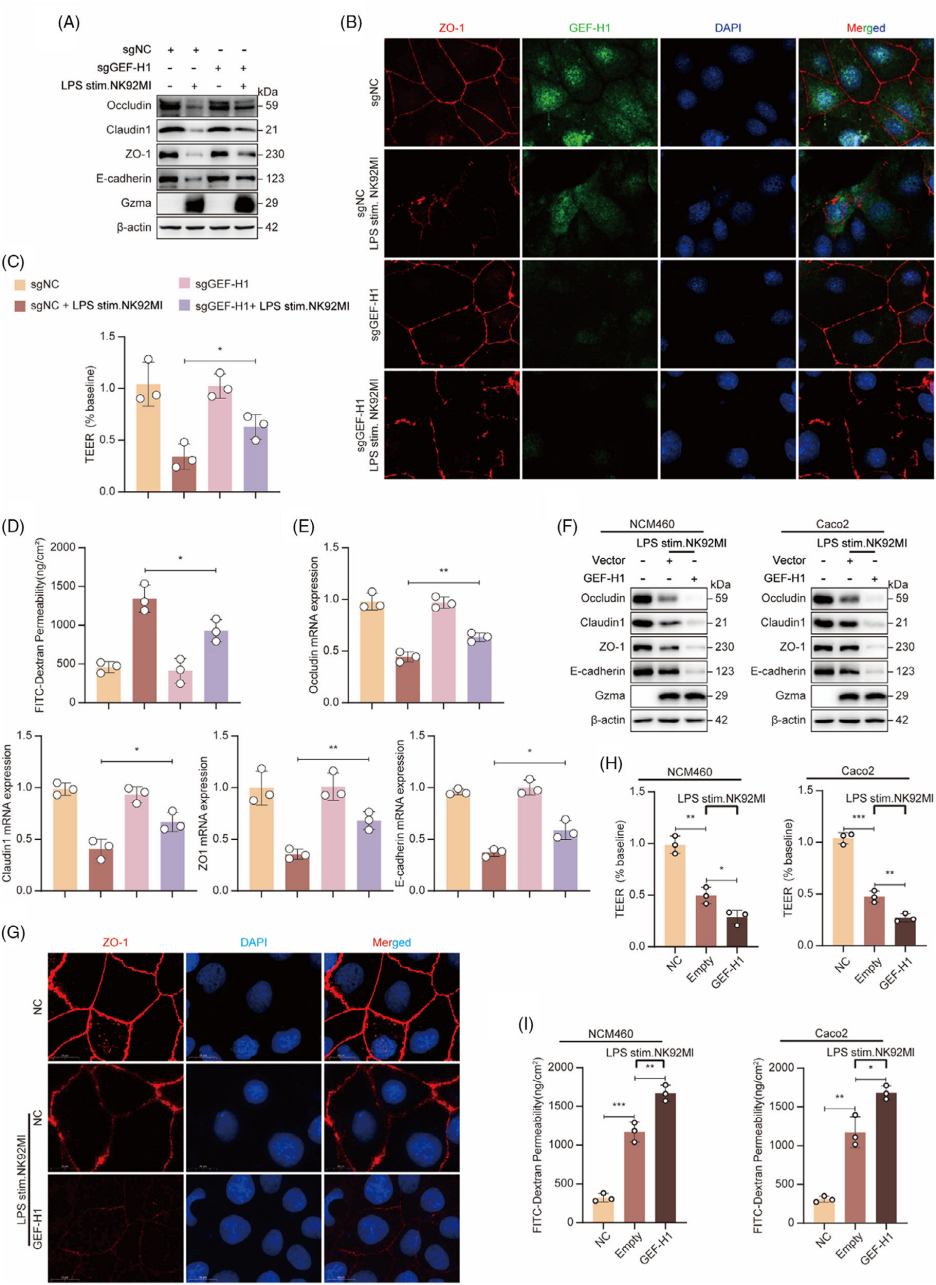
In vitro validation of GEF‐H1's essential role in Gzma‐mediated intestinal barrier injury during sepsis. (A) Western blot analysis of Occludin, Claudin‐1, ZO‐1, E‐cadherin, Gzma, and β‐actin (loading control) in NCM460 cells. Cells were transfected with sgNC (control) or sgGEF‐H1, followed by co‐culture with LPS‐stimulated NK92MI cells. (B) Immunofluorescence staining of ZO‐1 (red), GEF‐H1 (green), and DAPI (blue) in Caco2 cells under the same treatment conditions as (A). Scale bar: 20 µm. (C) TEER measurement of NCM460 cell monolayers transfected with sgGEF‐H1 or sgNC, then co‐cultured with LPS‐stimulated NK92MI cells (mean ± SEM, *n* = 3 per group; *p < .05, two‐way ANOVA). (D) FITC‐dextran permeability assay in NCM460 cell monolayers [same treatment as (C)]. Transfection with sgGEF‐H1 significantly reduced FITC‐dextran permeability compared with sgNC controls, further confirming improved barrier function upon GEF‐H1 depletion (mean ± SEM, *n* = 3 per group; *p < .05, two‐way ANOVA). (E) qPCR analysis of NCM460 cells showed that sgGEF‐H1 transfection reversed the transcriptional downregulation of Occludin, Claudin‐1, ZO‐1, and E‐cadherin induced by co‐culture with LPS‐stimulated NK92MI cells, relative to sgNC controls (mean ± SEM, *n* = 3 per group; *p < .05, **p < .01, two‐way ANOVA). (F) Western blot analysis of Occludin, Claudin‐1, ZO‐1, E‐cadherin, Gzma, and β‐actin (loading control) in NCM460 and Caco2 cells. Cells were transfected with the GEF‐H1 overexpression vector or the empty vector (control), followed by co‐culture with LPS‐pretreated NK92MI cells. (G) Immunofluorescence staining of ZO‐1 (red) and DAPI (blue) in NCM460 cells after GEF‐H1 overexpression and co‐culture with LPS‐pretreated NK92MI cells (scale bar = 20 µm). (H) TEER measurement of NCM460 and Caco2 cells overexpressing GEF‐H1 or empty vector (control), following co‐culture with LPS‐pretreated NK92MI cells (mean ± SEM, *n* = 3/group; *p < .05, **p < .01, ***p < .001, one‐way ANOVA). (I) FITC‐dextran permeability assay in NCM460 and Caco2 cells transfected with GEF‐H1 overexpression vector or empty vector (control), then co‐cultured with LPS‐pretreated NK92MI cells (mean ± SEM, *n* = 3/group; *p < .05, **p < .01, ***p < .001, one‐way ANOVA).

To gain deeper insights into how GEF‐H1 modulates cellular signalling pathways in intestinal epithelial barrier function, a comprehensive set of in vitro cellular assays was conducted. Initially, in the NCM460 cell, targeted knockout of GEF‐H1 was achieved via transfection with sgGEF‐H1, followed by co‐culture with LPS‐pretreated NK92MI cells. WB analysis demonstrated that, unlike cells transfected with the negative control sgNC, sgGEF‐H1 transfection markedly reversed the immune cell‐induced downregulation of proteins associated with tight junctions (including occludin, claudin‐1, and ZO‐1) as well as E‐cadherin, a key component of adherens junctions (Figure 5A). Immunofluorescence staining further validated that sgGEF‐H1 transfection significantly restored the continuous distribution and membrane localization of ZO‐1 in intestinal epithelial cells (Figure 5B). Assessment of core barrier function indicators revealed that knocking down GEF‐H1 resulted in a marked elevation of TEER across the cellular monolayer and a pronounced decrease in the paracellular flux of FITC‐labelled dextran (Figure 5C,D). Moreover, mRNA level detection confirmed that sgGEF‐H1 transfection also effectively reversed the downregulation of the aforementioned junction proteins at the transcriptional level (Figure 5E). Collectively, these results underscore that GEF‐H1 knockout can effectively enhance the structural integrity and functional stability of the intestinal epithelium's protective barrier.

To further consolidate the regulatory role of GEF‐H1, primary intestinal epithelial cells with GEF‐H1^−/−^ and NCM460 cells with Tet‐on system‐mediated conditional overexpression of GEF‐H1 were utilized, and the aforementioned barrier‐related indicators were re‐evaluated. Consistent results were obtained: downregulation of GEF‐H1 expression markedly enhanced both the structural stability and functional performance of the intestinal epithelial barrier, whereas its overexpression exacerbated barrier damage (Figure S4A–K). This further confirms that GEF‐H1 exerts an inhibitory influence on the structural soundness and physiological performance of the gut epithelial barrier.

In NCM460, which exhibits normal physiological characteristics, systematic optimization of transfection conditions for GEF‐H1‐specific siRNA was conducted. The results showed that treating cells with 50 nmol/L siRNA for 36 h efficiently downregulated GEF‐H1 expression (Figure S5A). This optimized protocol was adopted for all subsequent related experiments to ensure the stability and reliability of the gene silencing effect.

To clarify the universality of GEF‐H1's regulatory effect, GEF‐H1 was knocked down in three intestinal epithelial cell models (Caco2, HT‐29, and NCM460), which were then co‐cultured with LPS‐pretreated NK92MI cells. The findings revealed that silencing GEF‐H1 effectively restored the expression levels of key junctional proteins in intestinal epithelial cells, both at the protein and mRNA levels. These included the tight junction proteins occludin, claudin‐1, and ZO‐1, as well as the adherens junction protein E‐cadherin (Figure S5B,C). Immunofluorescence staining further revealed that GEF‐H1 knockdown substantially restored the continuous distribution and membrane localization of ZO‐1 and E‐cadherin (Figure S5D). Barrier function assessment showed that TEER values were significantly increased, while FITC‐dextran permeability was markedly decreased in all three cell models (Figure S5E,F), confirming the cell type‐independent regulatory effect of GEF‐H1 on the intestinal epithelial barrier. From a mechanistic perspective, Western blotting demonstrated that knockdown of GEF‐H1 significantly inhibited the phosphorylation of critical downstream targets in the RhoA/ROCK pathway—namely, LIMK, MLC2, and cofilin (Figure S5G,H). This implies that GEF‐H1 could contribute to immune cell–triggered breakdown of the intestinal epithelial barrier by activating the RhoA/ROCK signalling cascade.

To confirm the functional consequences of GEF‐H1 overexpression, GEF‐H1 was overexpressed in the aforementioned intestinal epithelial cells, which were then co‐cultured with LPS‐pretreated NK92MI cells. The results showed that GEF‐H1 overexpression further exacerbated LPS‐induced epithelial barrier damage, as evidenced by the significant downregulation of Occludin, Claudin1, ZO‐1, and E‐cadherin at both the protein and translational levels (Figure 5F; Figure S6A,B). Immunofluorescence staining revealed more pronounced disruption of the membrane continuity of ZO‐1 and E‐cadherin (Figures 5G; Figure S6C). Functionally, GEF‐H1 overexpression resulted in decreased TEER values and increased FITC‐dextran permeability (Figures 5H,I; Figure S6D,E), further validating the compromised integrity of the epithelial barrier. Moreover, there was a marked upregulation in the activation of key proteins associated with the RhoA/ROCK signalling cascade—including RhoA itself, p‐LIMK, p‐MLC2, and p‐cofilin (Figure S6F,G), which further supports the conclusion that GEF‐H1 disrupts epithelial barrier integrity by enhancing the RhoA–ROCK signal transduction cascade.

To conclude, our results reveal that Gzma triggers the activation of GEF‐H1, resulting in the subsequent regulation of the RhoA/ROCK signalling pathway and ultimately influencing intestinal epithelial barrier integrity. Using in vitro models, this work establishes GEF‐H1 as a critical mediator in Gzma‐induced disruption of the gut epithelial barrier under septic conditions. These findings underscore GEF‐H1's promise as a novel therapeutic intervention to safeguard intestinal barrier function during sepsis.

### 2.6 Mutations at the GEF‐H1 S886 functional site inhibit intestinal epithelial barrier injury

**FIGURE 6:**
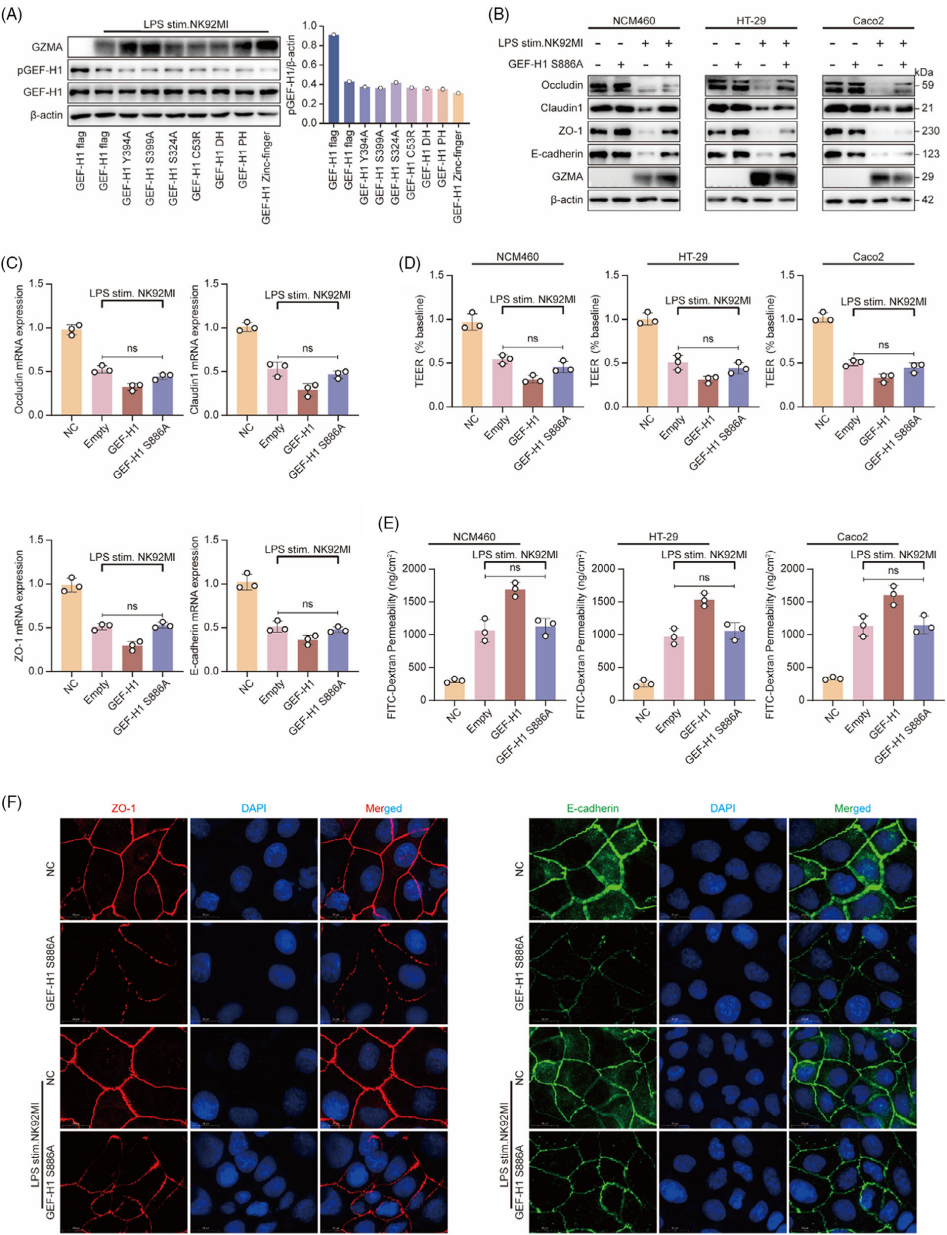
Mutations at the GEF‐H1 S886 functional site inhibit intestinal epithelial barrier injury. (A) Western blot analysis of GEF‐H1 S886 phosphorylation levels (left panel, with β‐actin as the loading control) and corresponding grayscale quantification (right panel) in human colonic epithelial NCM460 cells co‐cultured with LPS‐pretreated NK92MI cells following transfection with various GEF‐H1 domain or phosphorylation site mutants. (B) Western blot detection of Occludin, Claudin1, ZO‐1, and E‐cadherin in human colonic epithelial cells (NCM460, HT‐29, Caco2) co‐cultured with LPS‐pretreated NK92MI cells after transfection with the GEF‐H1 S886A mutant (β‐actin as loading control). (C) RT‐qPCR analysis of mRNA expression levels of Occludin, Claudin1, ZO‐1, and E‐cadherin in NCM460 cells co‐cultured with LPS‐pretreated NK92MI cells following transfection with the GEF‐H1 S886A mutant (mean ± SEM, *n* = 3/group; one‐way ANOVA; ns: not statistically significant). (D) TEER measurements in NCM460, HT‐29, and Caco2 cells co‐cultured with LPS‐pretreated NK92MI cells after transfection with the GEF‐H1 S886A mutant (mean ± SEM, *n* = 3/group; one‐way ANOVA; ns: not statistically significant). (E) FITC‐dextran permeability assays in NCM460, HT‐29, and Caco2 cells co‐cultured with LPS‐pretreated NK92MI cells after transfection with the GEF‐H1 S886A mutant (mean ± SEM, *n* = 3/group; ns: not statistically significant). (F) Immunofluorescence staining of ZO‐1 (left, red) and E‐cadherin (right, green) in NCM460 cells co‐cultured with LPS‐pretreated NK92MI cells following transfection with the GEF‐H1 S886A mutant (blue: DAPI nuclear staining; scale bar = 20 µm).

We have confirmed that Gzma can activate the GEF‐H1 protein by mediating the dephosphorylation of S886. However, it remains unclear whether Gzma regulates GEF‐H1 activity exclusively through this site. To further explore whether GEF‐H1 contains other key structural domains or phosphorylation sites involved in its functional regulation, we transfected expression plasmids encoding various GEF‐H1 domain deletion mutants and phosphorylation site mutants into NCM460 cells. The findings revealed a notable difference when contrasted with the control group; mutations at other sites or structural domains did not significantly alter the phosphorylation level of GEF‐H1 at S886 (Figure 6A). This suggests that S886 is likely a critical target through which Gzma regulates GEF‐H1 activity.

To further validate the biological relevance of GEF‐H1 S886 phosphorylation site, we transfected a GEF‐H1 S886 site mutant (S886A) into three intestinal epithelial cell lines—NCM460, Caco2, and HT‐29—and co‐cultured these with LPS‐pretreated NK92MI cells. The findings revealed that the S886 mutation markedly enhanced both the protein and transcription of critical tight junction components—namely, Occludin, Claudin‐1, ZO‐1—as well as E‐cadherin, in all three tested cell lines (Figure 6B,C). Moreover, the mutation partially recovered the TEER of the epithelial cells and markedly reduced FITC‐dextran intestinal permeability (Figure 6D,E). Immunofluorescence staining further revealed that the mutation enhanced the continuous distribution and membrane localization integrity of ZO‐1 and E‐cadherin (Figure 6F), suggesting a partial restoration of epithelial barrier structure and function.

In summary, these findings indicate that Gzma primarily exerts its detrimental effect on epithelial barrier integrity via modulation of GEF‐H1 dephosphorylation at Ser886. Moreover, mutation at this specific site can partially restore epithelial barrier integrity compromised by Gzma, thereby further establishing S886 as a key regulatory residue within the Gzma/GEF‐H1 signalling pathway.

### 2.7 Systematic screening and validation of GEF‐H1 modulator in small molecule compound libraries

Next, we conducted an in‐depth investigation into the precise activation mechanism of GEF‐H1 through a systematic screening of a targeted small‐molecule compound libraries. Full activation of GEF‐H1 depends on a tightly regulated two‐step process: first, GEF‐H1 is released from microtubules during cytoskeletal dynamic remodelling (such as microtubule depolymerization), which induces a conformational change that exposes its PH domain.^30^ Subsequently, this domain recognizes and binds to the phospholipid PIP_2_ on the cell membrane, thereby recruiting GEF‐H1 to the membrane region where its substrate Rho protein resides, facilitating essential membrane localization.^31^ The second step involves dephosphorylation at the S886 residue, which triggers a conformational rearrangement within the carboxyl‐terminal domain, thereby fully relieving the autoinhibition of the adjacent DH domain. This enables membrane‐localized GEF‐H1 to efficiently interact with and activate RhoA, thereby initiating downstream signalling pathways.^32^

**FIGURE 7:**
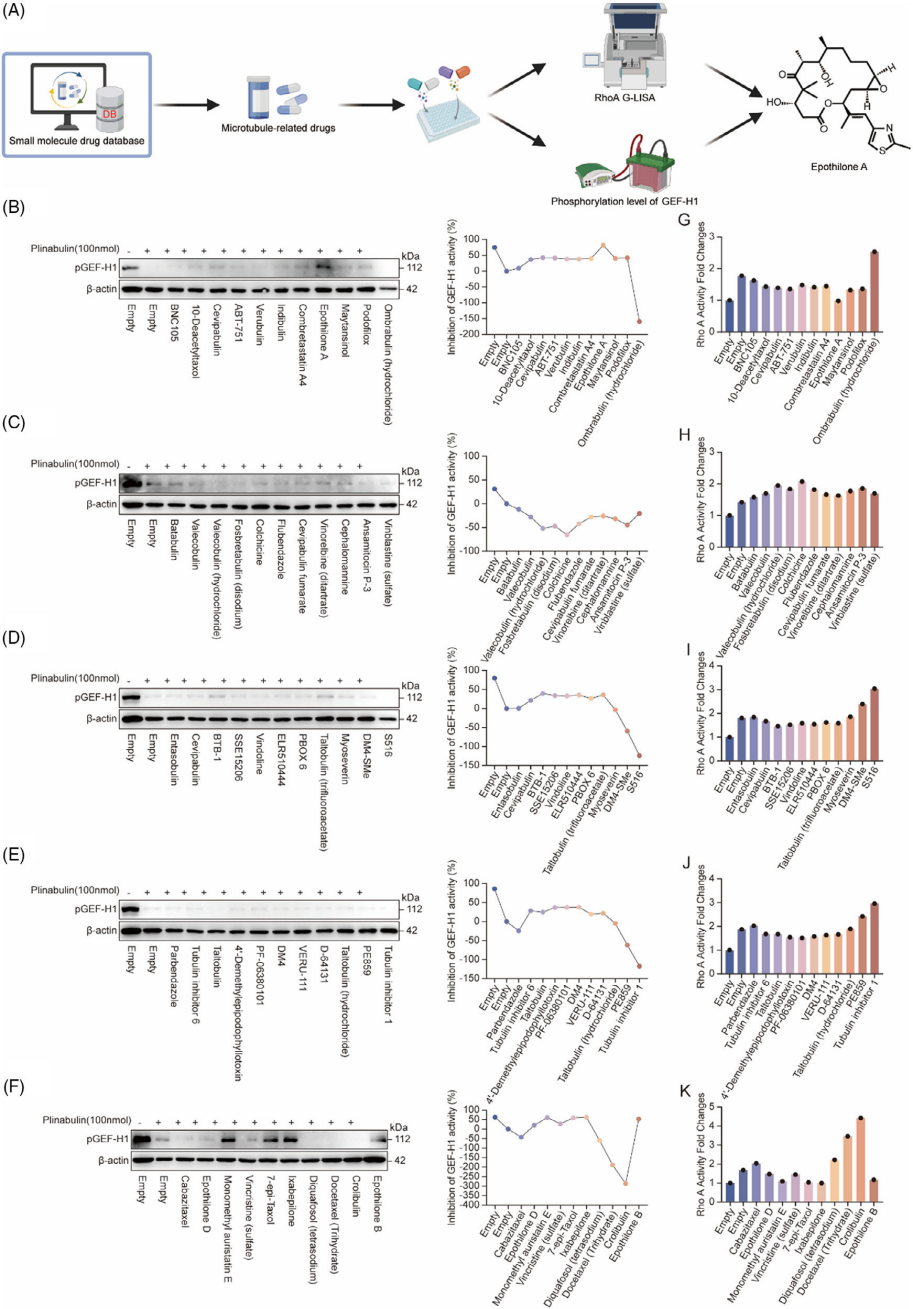
Systematic screening and validation of GEF‐H1 modulator in small molecule compound libraries. (A) Schematic diagram depicting the workflow for screening GEF‐H1 modulator, which includes sequential steps of structure‐based molecular docking, virtual screening, RhoG‐LISA validation, and Western blot verification. The chemical structure of the representative compound Epothilone A is displayed on the right. (B–F) Western blot analysis of p‐GEF‐H1 (S886) and β‐actin expression levels (left panel) in NCM460 cells treated with various microtubule‐targeting compounds, accompanied by corresponding graphs illustrating the percentage inhibition of GEF‐H1 activity (right panel). (G–K) Bar graphs summarizing the fold changes in RhoA activity in NCM460 cells after treatment with different compounds.

Given the complexity and the large number of kinases responsible for modulating the phosphorylation level of GEF‐H1 at serine residue 886,^12^, ^30^, ^32^, ^33^, ^34^ we focused our screening efforts on microtubule‐stabilizing agents. These compounds are capable of inhibiting microtubule depolymerization, thus inhibiting GEF‐H1 dissociation from microtubules and suppressing its later recruitment to the plasma membrane, ultimately interfering with its activation. From the compound library, we initially selected 54 microtubule‐targeting drugs, including Epothilones, Taxol derivatives, and Vinca alkaloids such as Epothilone A and Docetaxel. Using the microtubule‐depolymerizing agent Plinabulin—which acts as a GEF‐H1 agonist—serving as a positive control, we assessed the suppressive actions of these compounds on GEF‐H1 activity in the NCM460 cell model. This was achieved by measuring the fold change in RhoA activity and analyzing GEF‐H1 phosphorylation levels via Western blotting (Figure 7A).

The findings from the experiments revealed that, in contrast to the control group, Epothilone A exerted the most pronounced inhibitory effect on GEF‐H1 activity, followed by Ixabepilone, Monomethyl auristatin E, 7‐epi‐Taxol, and Epothilone B. These compounds exhibited robust inhibition of GEF‐H1, whereas others, such as Maytansinol, displayed weaker inhibitory effects, with some showing almost no impact on GEF‐H1 activity (Figure 7B–F). To further validate these observations, we employed the G‐LISA assay to assess changes in RhoA activity. Compounds exhibiting strong GEF‐H1 inhibition, such as Epothilone A and Ixabepilone, were associated with a marked decrease in RhoA activity, as indicated by fold changes below 1. This pattern correlated well with the alterations in pGEF‐H1 expression observed via Western blot analysis (Figure 7G–K), suggesting that these microtubule‐stabilizing agents effectively inhibit the signalling pathway's activation involving GEF‐H1 and RhoA.

In summary, these results, derived from systematic screening, identify multiple microtubule‐stabilizing agents that inhibit the signalling cascade involving GEF‐H1 and RhoA. They underscore the essential role of these compounds in modulating cytoskeletal dynamics and GEF‐H1 activation, thereby providing a valuable experimental basis for future drug development targeting this pathway, such as the identification of a highly potent GEF‐H1 modulator.

### 2.8 Epothilone A attenuates sepsis‐induced intestinal barrier injury by inhibiting GEF‐H1 activity via phosphorylation

**FIGURE 8:**
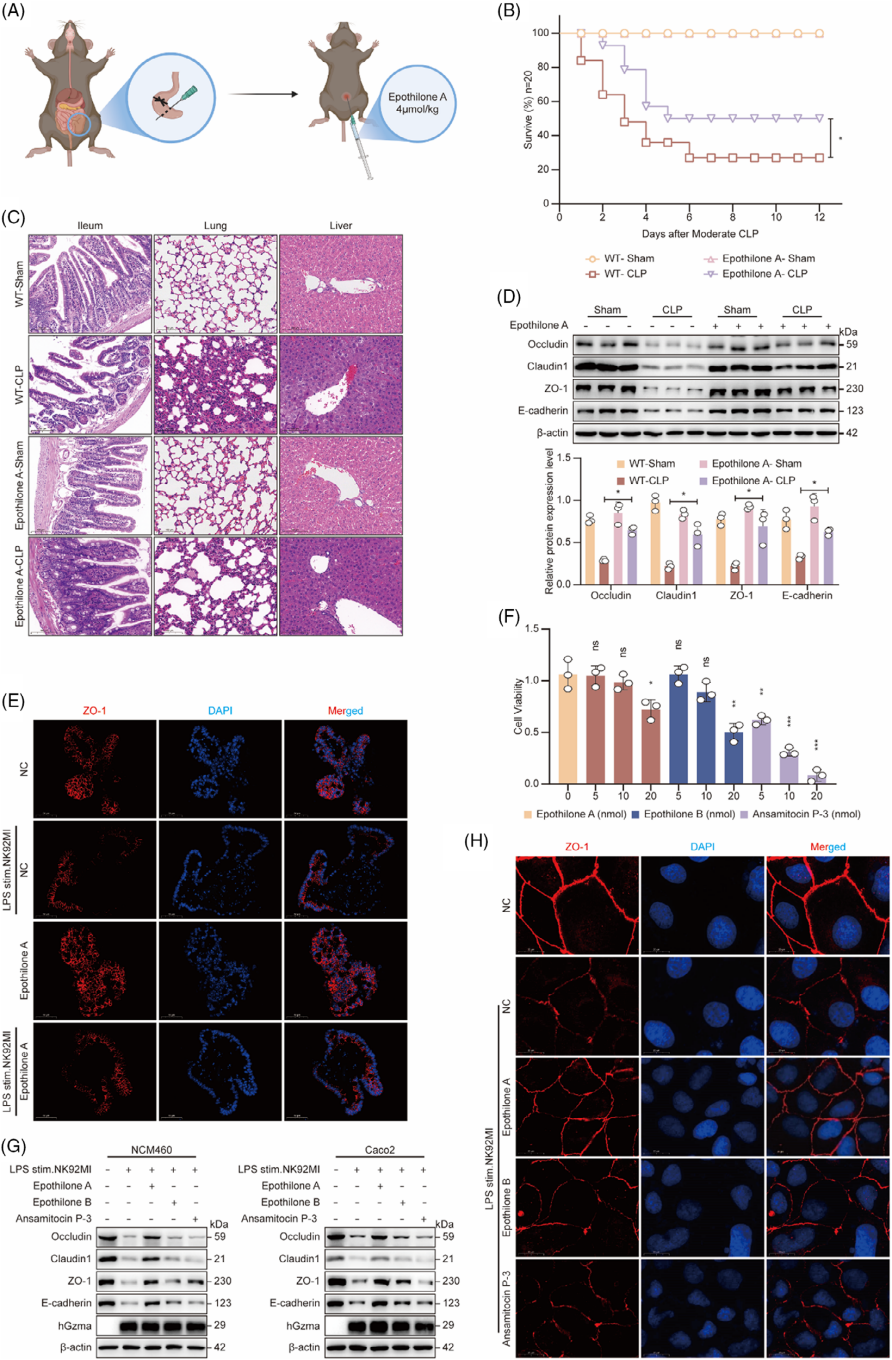
Epothilone A attenuates sepsis‐induced intestinal barrier injury by inhibiting GEF‐H1 activity via phosphorylation. Schematic diagram depicting the experimental timeline for the murine sepsis model and Epothilone A intervention, including the CLP procedure and intraperitoneal drug administration. (B) Survival curves of WT mice following sham operation, CLP, or CLP combined with Epothilone A treatment (*n* = 20 per group; *p < .05, Log‐rank test). (C) Histopathological analysis via HE staining of ileum, lung, and liver tissues from WT mice after sham, CLP, or CLP + Epothilone A treatment (scale bar = 100 µm). (D) Western blot analysis (upper panel) and corresponding grayscale quantification (lower panel, mean ± SEM, *n* = 3 per group) of tight junction proteins in ileal tissues of WT mice under sham, CLP, or CLP + Epothilone A treatment conditions (*p < .05, two‐way ANOVA). (E) Immunofluorescence staining of ZO‐1 in intestinal organoids after co‐culture with LPS‐stimulated NK92MI cells and Epothilone A intervention (nuclear staining: DAPI in blue; ZO‐1 in red; scale bar = 20 µm). (F) Cell viability of NCM460 cells assessed by CCK‐8 assay following treatment with varying concentrations of Epothilone A, Epothilone B, or Ansamitocin P‐3 (mean ± SEM, *n* = 3 per group; *p < .05, **p < .01, ***p < .001, Student's *t*‐test). (G) Western blot analysis of Occludin, Claudin1, ZO‐1, and E‐cadherin in human colonic epithelial cells (NCM460 and Caco2) after co‐culture with LPS‐stimulated NK92MI cells and treatment with Epothilone A, Epothilone B, or Ansamitocin P‐3 (β‐actin served as the loading control). (H) Caco2 cells were treated and co‐cultured as above. Immunofluorescence staining visualized ZO‐1 (red) and nuclei (blue, DAPI; scale bar = 20 µm).

To systematically evaluate the therapeutic efficacy and safety of Epothilone A in a sepsis model, we conducted a comprehensive set of experiments conducted both in living organisms and in controlled laboratory settings. Building upon previous findings by Eric Kratzer et al.,^35^ who demonstrated that Epothilone B exerts protective effects through the inhibition of GEF‐H1 activity and established its effective dosage in animal models (4 µmol/kg), we applied the same dosage for the preliminary assessment of Epothilone A's efficacy. In a mouse model of sepsis established through CLP, Epothilone A was delivered intraperitoneally immediately following model initiation. (Figure 8A). Survival analysis indicated that Epothilone A significantly reduced the mortality rate in septic mice relative to those receiving vehicle control treatment (Figure 8B), suggesting substantial in vivo protective effects.

**FIGURE 9:**
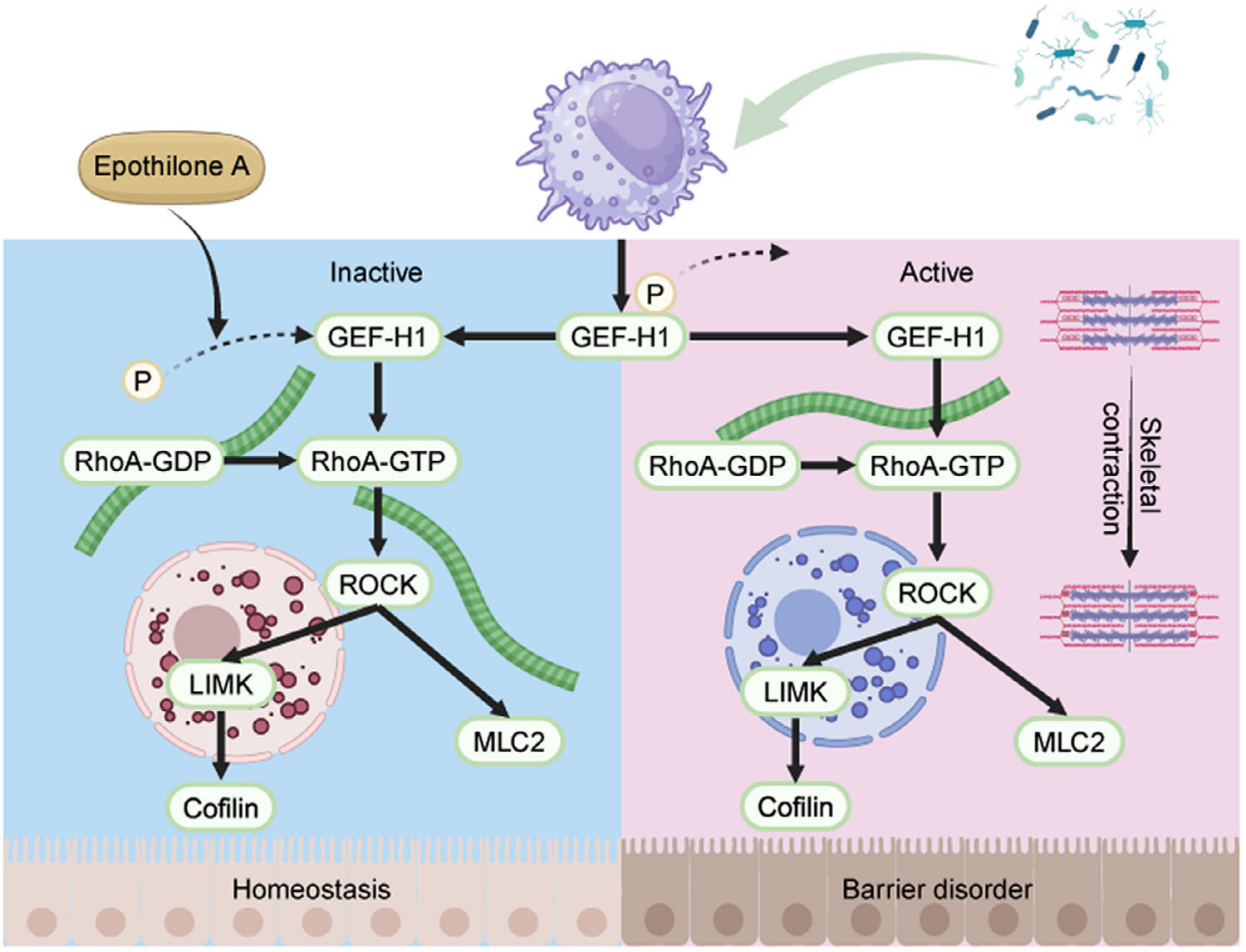
Epothilone A maintains intestinal barrier integrity by inhibiting GEF‐H1 to regulate the RhoA/ROCK pathway. Under physiological conditions (homeostasis), GEF‐H1 is maintained in an inactive, phosphorylated state, while RhoA remains bound to GDP. This arrangement keeps downstream effectors, including ROCK, LIMK, MLC2, and Cofilin, in an inactive form; thus, supporting a stable intestinal epithelial cytoskeleton and normal barrier function. However, during sepsis, immune‐cell‐derived Gzma induces the dephosphorylation of GEF‐H1 at Ser886. This activation promotes the formation of RhoA‐GTP, which subsequently activates ROCK. Activated ROCK then phosphorylates LIMK, MLC2, and Cofilin—resulting in cytoskeletal contraction and disruption of tight junctions that ultimately lead to barrier dysfunction. Treatment with Epothilone A stabilizes microtubules and inhibits GEF‐H1 activation; thereby suppressing the RhoA/ROCK pathway and preventing cytoskeletal hypercontraction while restoring epithelial barrier integrity during sepsis.

To further evaluate the protective effects of Epothilone A against organ injury, we conducted a comprehensive histopathological analysis. The results demonstrated that Epothilone A treatment markedly attenuated sepsis‐induced multi‐organ damage. In the intestinal tissues, compared with WT septic mice, the treated group displayed a more intact intestinal mucosal architecture, well‐organized villi, improved epithelial continuity, and reduced inflammatory cell infiltration in the lamina propria. In the lungs, Epothilone A significantly ameliorated alveolar structural disruption, interstitial oedema, and accumulation of inflammatory cells. In the liver, the compound effectively alleviated pathological alterations such as hepatocyte necrosis, vacuolar degeneration, and sinusoidal congestion (Figure 8C).

To gain deeper insights into how Epothilone A safeguards intestinal barrier integrity, we quantified the protein and mRNA expression of key tight junction proteins and cell adhesion molecules via Western blotting and qPCR. The findings revealed that administering Epothilone A markedly enhanced the intestinal tissue concentrations of critical tight junction proteins—including Occludin, Claudin‐1, and ZO‐1—along with E‐cadherin (Figure 8D). These results indicate that Epothilone A exerts its protective action through enhancing and reinstating the expression of critical molecules involved in maintaining epithelial barrier integrity.

To verify whether Epothilone A acts by inhibiting the Gzma‐mediated GEF‐H1 activation pathway, we established a co‐culture system of LPS‐pretreated NK92MI cells and intestinal organoids, and subsequently treated the system with Epothilone A. Immunofluorescence staining revealed that Epothilone A treatment effectively preserved the continuity and membrane localization of the ZO‐1 protein, thereby significantly attenuating Gzma‐induced epithelial barrier damage (Figure 8E).

In in vitro cell models, we systematically evaluated the efficacy and safety of Epothilone A, using the previously reported Epothilone B and the initial screening negative control compound Ansamitocin P‐3 as references. Concentration‐gradient experiments revealed that Epothilone A exerted the most pronounced inhibitory effect on GEF‐H1 at a concentration of 20 nmol, although this dosage caused a mild reduction in cell viability (Figure 8F; Figure S6A). Based on these findings, we selected a concentration of 10 nmol for subsequent experiments, with Epothilone B and Ansamitocin P‐3 tested in parallel at the same level.

The results demonstrated that Epothilone A treatment significantly increased the expression levels—both at the protein and mRNA levels—of key tight junction and adherens junction proteins, including Occludin, Claudin‐1, ZO‐1, and E‐cadherin, in intestinal epithelial cells (Figure 8G; Figure S6B,C). Functional analyses revealed that Epothilone A treatment markedly enhanced the TEER and decreased FITC‐dextran permeability, thereby confirming a notable improvement in epithelial barrier function (Figure S6D,E). Furthermore, immunofluorescence staining showed that Epothilone A facilitated the continuous distribution and membrane localization integrity of ZO‐1 and E‐cadherin (Figure 8H; Figure S6F).

Mechanistically, WB demonstrated that Epothilone A administration significantly suppressed the phosphorylation of critical downstream signalling molecules, including RhoA, LIMK, MLC2, and Cofilin, in the RhoA/ROCK signalling pathway (Figure S6G,H), suggesting its potential to mitigate sepsis‐induced epithelial barrier dysfunction through modulation of this pathway.

In summary, these findings demonstrate that Epothilone A effectively reduces multiple organ injuries, significantly improves intestinal barrier function, and enhances survival rates in sepsis models established both in living organisms and in laboratory settings. The compound's protective activity likely stems from its ability to suppress the Gzma/GEF‐H1/RhoA/ROCK signalling cascade while concurrently promoting the recovery of tight junction proteins and cell adhesion molecules. Collectively, these findings indicate that Epothilone A could serve as a promising candidate for sepsis treatment, especially in cases characterized by impaired epithelial barrier integrity.

## 3 DISCUSSION

This study identifies a previously unrecognized mechanism by which Gzma, a serine protease released by cytotoxic lymphocytes, contributes to intestinal epithelial barrier dysfunction during sepsis through direct activation of GEF‐H1 and subsequent engagement of RhoA/ROCK signalling. Elevated Gzma expression was observed in both clinical sepsis samples and experimental murine models, correlating with intestinal injury and systemic inflammation. Using in vitro co‐culture, genetic knockout, pharmacological inhibition, and structural biology approaches, we demonstrate that Gzma binds directly to GEF‐H1 and induces its dephosphorylation at S886, thereby triggering the RhoA/ROCK cascade. This activation promotes cytoskeletal reorganization, disruption of intercellular junctions, and subsequent intestinal barrier failure. These findings establish a functional correlation between the Gzma/GEF‐H1 axis and sepsis‐induced barrier disruption, with direct implications for therapeutic targeting.

The role of Gzma in intestinal barrier regulation presents a striking paradox. While our findings reveal a deleterious function in sepsis, a recent study by Niu et al. reported a protective effect in inflammatory bowel disease (IBD), wherein Gzma suppressed ferroptosis via the PDE4/cAMP/PKA/CREB signalling axis and upregulation of GPX4.^36^ This protective view is complicated by earlier reports where Müller et al.^38^ observed increased Gzma‐expressing cells in active IBD mucosa,^37^ and Santiago et al. found that Gzma knockout alleviated DSS‐induced colitis. Additionally, Scott et al.^39^ developed a chemiluminescence assay (Gzma‐CL) capable of detecting active Gzma in stool supernatants, noting elevated activity in IBD patients and showing that CD8^+^ T cell‐derived Gzma induced IL‐8 secretion via PAR1 signalling—a pro‐inflammatory mechanism inconsistent with a purely protective role. Several factors may help reconcile these conflicting observations. First, a critical mechanistic consideration highlighted by Zhong et al. is that Gzma functions as an intracellular protease requiring perforin‐mediated delivery to access cytosolic substrates.^40^ Without perforin, extracellular Gzma cannot engage canonical intracellular targets. This distinction may explain the protective effects reported by Niu et al.,^36^ where recombinant Gzma was applied in the absence of perforin, thus remaining extracellular and unable to access intracellular substrates like GEF‐H1. Conversely, in our sepsis model and during physiological cytotoxic lymphocyte‐mediated killing, perforin‐dependent delivery enables Gzma to access intracellular GEF‐H1, leading to barrier disruption. Second, the cellular origin of Gzma may determine its functional outcome: in our sepsis model, Gzma is derived from broadly activated cytotoxic lymphocytes during systemic inflammation,^41^ whereas Niu et al. implicated a regulatory CD8^+^CD39^+^ T cell subset whose CD39 expression contributes to immunoregulation via adenosine generation.^36^ Third, the inflammatory context differs substantially—sepsis represents an acute systemic hyperinflammatory state, while IBD involves compartmentalized T‐cell‐driven chronic inflammation with complex cycles of injury and repair. We propose that under acute severe inflammation, Gzma primarily exerts its proteolytic activity by targeting junctional regulators such as GEF‐H1, while in chronic settings, alternative substrates or interacting partners may direct Gzma towards different signalling pathways.

Consistent with our results, prior research has established GEF‐H1 as a central signalling hub in innate immunity.^42^ Zhao and colleagues demonstrated that GEF‐H1 facilitates detection of muramyl dipeptide by activating IKKε and phosphorylating IRF5.^43^ Kashyap et al.^44^ reported that microtubule‐disrupting chemotherapeutics release GEF‐H1 from microtubules, enhancing CD8^+^ T cell immunity. Our study extends this framework by identifying Gzma as a novel immune‐derived activator of GEF‐H1 during sepsis‐associated barrier disruption. The downstream consequences include sustained activation of RhoA and its effectors—ROCK, LIMK, MLC2, and cofilin—culminating in actomyosin contraction and disassembly of intercellular junctions, in line with established RhoA/ROCK signalling literature.^28^, ^29^, ^30^ From a therapeutic perspective, we identified microtubule‐stabilizing agents, particularly Epothilone A, as potent inhibitors of GEF‐H1 activation. Administration of Epothilone A in septic mice improved survival, reduced organ damage, and restored junctional protein expression. This strategy is grounded in the established model of microtubule‐mediated GEF‐H1 regulation: microtubule stabilization enhances GEF‐H1 sequestration, counteracting Gzma‐induced release. Our findings thus provide functional validation of this model in a clinically relevant context and establish proof‐of‐concept for targeting the Gzma/GEF‐H1/RhoA pathway in sepsis.

Several limitations warrant consideration. First, our in vitro model using LPS‐stimulated NK cells does not fully capture the complexity of the septic microenvironment. Second, the precise mechanism by which Gzma induces GEF‐H1 dephosphorylation remains unclear. As a serine protease, Gzma may act indirectly—for instance, by activating a phosphatase targeting GEF‐H1.^25^ Multiple phosphatases are known to regulate GEF‐H1 activity,^10^ and it is plausible that a class of phosphatases, rather than a single enzyme, participates in this process. Identifying the specific phosphatase(s) involved would require extensive screening beyond the current scope. Nevertheless, while elucidating the underlying mechanism remains important, the absence of an identified phosphatase does not diminish our core conclusion: targeting the Gzma/GEF‐H1 interaction holds therapeutic potential in sepsis. Third, the efficacy and safety of Epothilone A require further validation in higher organisms. Future studies should investigate the temporal dynamics of Gzma release and GEF‐H1 activation across sepsis stages, evaluate the contributions of other granzymes, and explore combination therapies. Patient‐derived organoids or humanized mouse models may offer more clinically relevant platforms. To specifically address the Gzma paradox, subsequent work should systematically compare Gzma's effects across disease models, identify disease‐specific interacting partners, and determine whether modulation of the Gzma/GEF‐H1 axis in IBD models recapitulates the protective effects observed by Niu et al.^36^

Building on structural insights from Zhong et al.,^40^ future investigations should rigorously characterize the oligomeric state of Gzma preparations used in different experimental systems. Similarly, the Gzma‐CL assay developed by Scott et al.^39^ offers a valuable tool for longitudinally monitoring active Gzma in patient biosamples. More broadly, integrating the structural framework of microtubule‐mediated GEF‐H1 regulation, future research should explore how tubulin post‐translational modifications—such as acetylation or detyrosination—modulate the sensitivity of the microtubule–GEF‐H1 axis to Gzma‐mediated disruption during sepsis. Understanding these regulatory layers may reveal additional therapeutic targets.

In conclusion, this study identifies the Gzma/GEF‐H1/RhoA signalling axis as a critical mechanistic link connecting immune activation with epithelial barrier dysfunction in sepsis (Figure 9). By integrating the established model of microtubule‐mediated GEF‐H1 sequestration—which describes how microtubules retain GEF‐H1 via its C1 domain—our work reveals how this homeostatic mechanism is pathologically subverted during sepsis through Gzma‐induced GEF‐H1 release. These findings deepen our understanding of sepsis pathophysiology and provide a foundation for developing interventions aimed at preserving intestinal barrier integrity. The contrasting roles of Gzma in sepsis‐induced barrier disruption versus IBD‐associated protection highlight the profound context‐dependency of granzyme function. Rather than representing a contradiction, this duality reflects the evolutionary versatility of immune proteases. Harnessing this duality for therapeutic benefit will require precise, context‐aware strategies that selectively amplify protective pathways while minimizing deleterious effects.

## 4 METHODS AND MATERIALS

### 4.1 Mice

All mice were maintained in a SPF environment. The GEF‐H1 knockout (GEF‐H1^−/−^) mouse strain was provided by Nanjing Medical University. Animals were group‐housed with their same‐litter siblings, with four to five individuals per cage, under standardized 12 h light/dark conditions. All animal‐related experimental procedures were reviewed and authorized approved by the Institutional Animal Care and Use Committee (IACUC) at Nanjing Medical University.

### 4.2 Cell culture

NCM460 cells were grown in RPMI1640 medium (G4532, Servicebio), supplemented with 10% (v/v) FBS (10099141, Gibco) and 100 U/mL penicillin–streptomycin (15140122, Gibco). Caco‐2 and HT‐29 cells were propagated in Dulbecco's Modified Eagle Medium (DMEM; G4512, Servicebio), also supplemented with 10% FBS and 100 U/mL penicillin–streptomycin. NK‐92MI cells were maintained in commercially available NK‐92 Complete Medium (CM‐0530, Procell). Mycoplasma testing confirmed the absence of contamination across all cell lines, and their genetic authenticity was verified via short tandem repeat (STR) analysis. The cultures were maintained under controlled environmental conditions: a temperature of 37°C, an atmosphere containing 5% CO_2_, and elevated humidity levels.

### 4.3 Antibodies and reagents

The antibodies used were anti‐GEF‐H1 (ab155785, Abcam), anti‐phospho‐GEF‐H1 (phosphorylated at Ser886, ab74156, Abcam), anti‐Gzma (ab209205, Abcam), anti‐Cofilin (10960‐1‐AP, Proteintech), anti‐MLC2 (10906‐1‐AP, Proteintech), anti‐LIMK1 (19699‐1‐AP, Proteintech), anti‐Claudin1 (13050‐1‐AP, Proteintech), anti‐E‐cadherin (20874‐1‐AP, Proteintech), anti‐Occludin (66378‐1‐Ig, Proteintech), anti‐ZO‐1 (21773‐1‐AP, Proteintech), anti‐phospho‐Cofilin (sc‐365882, Santa Cruz), anti‐phospho‐LIMK1 (phosphorylated at Thr508, #3841, Cell Signaling Technology), anti‐phospho‐MLC2 (phosphorylated at Thr18/Ser19, #3674, Cell Signaling Technology), and anti‐β‐actin (AC026, Abclonal). All antibodies were prepared at the concentrations recommended by their respective manufacturers. The following chemical substances were additionally employed: Plinabulin (HY‐14444, MedChemExpress), Epothilone A (HY‐13503, MedChemExpress), Epothilone B (HY‐17029, MedChemExpress), Ansamitocin P‐3 (HY‐15739, MedChemExpress), lipopolysaccharide (LPS) (Sigma‐Aldrich, L2630), FITC‐Dextran (MW 4000) (HY‐128868A, MedChemExpress), and Gzma (ab157288, Abcam).

### 4.4 The CLP mouse model

Sepsis was experimentally triggered in mice using the CLP model. Briefly, animals were rendered unconscious with anaesthesia, followed by a small longitudinal incision along the midline of the abdomen. The cecum was carefully exposed, and roughly 75% of its distal segment was tied off with 3‐0 silk suture—taking care to avoid compromising luminal continuity. An 18‐gauge needle was then used to perforate the ligated cecum, and a minimal quantity of faecal content was gently extruded through the puncture to verify patency. Subsequently, the cecum was returned to its anatomical position within the peritoneal cavity, and the abdominal wound was sutured closed. In contrast, sham‐operated control mice underwent identical surgical exposure and handling of the cecum, but without the need for suturing or piercing. All animals received a subcutaneous injection of.3 mL PBS immediately after surgery to support hemodynamic stability. At predetermined temporal points, mice were humanely euthanized, and tissues—including intestinal segments, liver, lung, and peripheral blood were harvested. Plasma was isolated from anticoagulant‐treated whole blood via centrifugation at 4°C (3000×*g*, 10 min) and reserved for flow cytometric profiling. Intestinal specimens were rapidly dissected into small fragments, snap‐immersed in liquid nitrogen and rapidly cooled, and stored at –80°C. Additional tissue samples (intestine, liver, and lung) were fixed in 4% PFA, embedded, and prepared for histopathological evaluation. Mortality was tracked continuously for 12 days following the procedure.

### 4.5 RNA isolation and real‐time quantitative PCR

Genomic DNA was isolated from 200 µL of plasma samples employing the QIAamp DNA Blood Mini Kit (Qiagen), strictly adhering to the supplier's recommended protocol. For RNA isolation, cultured cells or tissue specimens were processed via TRIzol‐based extraction. Specifically, each sample was uniformly suspended in 1 mL of TRIzol reagent, and then 200 µL of chloroform was added. After vigorous mixing and phase separation via centrifugation (12 000×*g*, 4°C, 15 min), the water‐based phase was carefully collected and mixed with 500 µL isopropanol. Precipitation proceeded at room temperature for 10 min, after which RNA was isolated via centrifugal precipitation (12 000×*g*, 10 min). The pellet was rinsed once using 70% ethanol (7500×*g*, 5 min) and dissolved in RNase‐free water. The first‐strand cDNA was generated using the HiScript III RT SuperMix kit (R323, Vazyme). To assess circulating mitochondrial DNA levels, two mitochondrial genomic regions—D‐loop and ND2—were targeted. qPCR was performed on a QuantStudio 3 Real‐Time PCR System (Applied Biosystems) with SYBR Green‐based qPCR Master Mix (Cat. No. Q711, Vazyme Biotech), following the vendor's guidelines. Each specimen was analyzed in duplicate, with three technical replicates. Expression data were normalized to β‐actin mRNA levels, and quantitative comparison between samples was determined using the 2^−ΔΔC^ *^t^* method. Data are presented as the ratio of treated group values to those of the untreated control group, arbitrarily assigned a value of 1. Primers were as follows: IFN‐β forward, 5′‐ccctatggagatgacggaga‐3′, and reverse, 5′‐ctgtctgctggtggagttca‐3′; IL‐6 forward, 5′‐ctgatgctggtgacaaccac‐3′, and reverse, 5′‐tccacgatttcccagagaac‐3′; TNFα forward, 5′‐tagccaggagggagaacaga‐3′, and reverse, 5′‐ttttctggagggagatgtgg‐3′; GAPDH forward, 5′‐aactttggcattgtggaagg‐3′, and reverse, 5′‐ggatgcagggatgatgttct‐3′; E‐cadherin forward, 5′‐cgagagctacacgttcacgg‐3′, and reverse, 5′‐gggtgcgagggaaaaatagg‐3′; ZO‐1 forward, 5′‐caacatacagtgacgcttcaca‐3′, and reverse, 5′‐cactattgacgtttccccactc‐3′; Claudin1 forward, 5′‐cctcctgggagtgatagcaat‐3′, and reverse, 5′‐ggcaactaaaatagccagacct‐3′; Occludin forward, 5′‐acaagcggttttatccagagtc‐3′, and reverse, 5′‐gtcatccacagggaagttaat‐3′.

### 4.6 Western blotting

Tissues and cultured cells were homogenized in RIPA lysis buffer (P0013B, Beyotime) supplemented with a cocktail of Proteolytic enzyme inhibitors (HY‐K0010, MedChemExpress) and freshly prepared 1 mM phenylmethylsulfonyl fluoride (PMSF; ST506, Beyotime; diluted from a 100 mM stock solution). To prevent dephosphorylation, phosphatase inhibitors (HY‐K0021, MedChemExpress) were introduced into the lysate to achieve the specified final concentration at a 1:100 dilution and incubated on ice for 20 min. The sample was then centrifuged at 12 000×*g* for 15 min at 4°C, followed by collection of the supernatant. An aliquot of the supernatant was combined with a 5× SDS sample buffer supplemented with β‐mercaptoethanol (sample‐to‐buffer ratio = 1:4, yielding a final 1× concentration), subsequently subjected to heat‐induced denaturation at 100°C for 10 min. Protein separation was carried out via SDS‐PAGE, after which proteins were electrotransferred onto polyvinylidene fluoride (PVDF) membranes (Millipore). Membranes were blocked for 1 h at room temperature in TBST (Tris‐buffered saline with.1% Tween‐20) containing 5% (w/v) non‐fat dry milk (i.e., 5 g dissolved in 100 mL TBST). Primary antibodies—diluted in antibody diluent according to manufacturer recommendations (typically 1:1000–1:5000)—were incubated overnight at 4°C. Following three rinses in TBST, the membranes were subsequently exposed to suitable secondary antibodies conjugated with horseradish peroxidase (HRP) for 1 h at ambient temperature (e.g., #7074 or #7076, Cell Signaling Technology), diluted at a ratio of 1:5000 in TBST containing 5% non‐fat dry milk. Immunoreactive bands were visualized using enhanced chemiluminescence (ECL) reagent (prepared by mixing equal volumes of stock solutions A and B), applying 200 µL per membrane strip, and identified via the Tanon 5200 Chemiluminescent Imaging Platform (Tanon, Shanghai, China). Quantitative densitometry was carried out with ImageJ software (version 1.49, National Institutes of Health, Bethesda, MD, USA).

### 4.7 RhoA activation assay

RhoA activation levels were quantified using the G‐LISA Assay for Quantifying RhoA Activity (Cytoskeleton, Inc., Denver, CO, USA; Cat. No. BK121), a luminescence‐based detection system. Per the protocol provided by the manufacturer, intestinal epithelial cells were first lysed to obtain whole‐cell protein extracts, and total protein concentration was measured accordingly. Samples were normalized to 1.5 mg/mL before assay. After incubating with the anti‐RhoA primary antibody for 45 min at ambient temperature, luminescent signals were recorded at an emission wavelength of 490 nm using a Luminoskan Ascent microplate reader (Thermo Scientific, USA; Model 5300173), following the supplier's instructions.

### 4.8 Histological analysis

Tissue specimens were preserved in 4% phosphate‐buffered formalin before histopathological examination and then processed for paraffin wax infiltration and embedding. HE staining was performed on 4 µm‐thick sections. Intestinal damage was quantified according to the Chui histopathological scoring criteria. Pulmonary inflammation and structural injury were systematically evaluated across the entire lung parenchyma, with assessment focusing on five key features: alveolar neutrophil infiltration, interstitial neutrophil accumulation, hyaline membrane formation, intra‐alveolar deposition of proteinaceous material, and thickening of alveolar septa. Each feature was graded semi‐quantitatively from 0 (no abnormality) to 2 (marked or widespread involvement). Hepatic inflammatory changes were scored based on four histological indicators: thrombus count, number of microabscesses or abscesses, degree and distribution of inflammatory cell infiltration, and extent of hepatocellular necrosis—each rated on a 0–3 scale (0 = absent; 3 = extensive or severe). All histological images were captured with a Nikon Eclipse 50i upright microscope equipped with digital imaging software.

### 4.9 Small interfering RNAs

The small interfering RNAs (siRNAs) employed in this investigation were commercially obtained from GENERAL BIOL (Chuzhou, China). Transfection of cultured cells with siRNA duplexes was performed using either Lipofectamine 3000 (Invitrogen) or INTERFERin (Polyplus‐transfection), strictly following the protocols provided by the respective manufacturers. The nucleotide sequences of all siRNAs utilized are listed below: h‐siGEF‐H1‐1, forward: 5′‐AGACAGAGGAUGAGGCUUATT‐3′, reverse: UAAGCCUCAUCCUCUGUCUTT; h‐siGEF‐H1‐2, forward: 5′‐GGACAAGCCUUCAGUGGUATT‐3′, reverse: UACCACUGAAGGCUUGUCCTT; h‐siGEF‐H1‐3, forward: 5′‐GUACCAAGGUCAAGCAGAATT‐3′, reverse: UUCUGCUUGACCUUGGUACTT.

### 4.10 GEF‐H1‐sgRNA

The sgRNA utilized in this study was purchased from Nanjing Keris Biotechnology Co., Ltd., and all experimental procedures were strictly followed according to the manufacturer's instructions. On the first day, target cells of good quality were seeded into 24‐well plates at a concentration of 3 × 10^5^ cells/mL, with 500 µL per well to achieve approximately 1.5 × 10^5^ cells per well; the seeding density could be adjusted based on cell growth rate to reach 30%–50% confluence the next day before viral infection. After seeding, the cells were cultured overnight at 37°C with 5% CO_2_. On the second day, viral infection was performed using the half‐volume method: the original medium was aspirated, 250 µL of fresh medium was added, and the virus was thawed on ice. The required virus volume was calculated as (MOI × cell number)/virus titre (TU/mL) × 1000, and polybrene was added if needed. Four hours post‐infection, the medium was replenished to 500 µL. On the third day, within 10 to 24 h post‐infection, the virus‐containing medium was replaced with fresh complete medium. From day four to five, infection efficiency was assessed: if it carried a puromycin resistance gene, medium containing an appropriate concentration of puromycin was added at 48 h to select for stably transduced cell lines. The sequence of the sgRNA used was sgGEF‐H1, with forward primer caccgAGAGGCTCACCCAGCAATGT and reverse primer aaacACATTGCTGGGTGAGCCTCTc.

### 4.11 Immunohistochemistry and immunofluorescence analyses

Immunohistochemistry (IHC) and immunofluorescence (IF) assays were carried out following established protocols. In summary, tissue specimens were fixed using a 4% paraformaldehyde solution and subsequently processed for paraffin embedding. Following sectioning, slides were subjected to overnight incubation at 4°C using suitable primary antibodies, and subsequently treated with corresponding secondary antibodies. For IHC, antigen visualization was performed using the horseradish peroxidase (HRP)‐mediated chromogenic reaction with 3,3′‐diaminobenzidine (DAB), yielding a distinct brown signal in positive cells. In IF experiments, nuclear counterstaining was performed using DAPI (Cat. No. G1012, Servicebio). After air‐drying, sections were sealed with ProLong Antifade Mountant (Cat. No. G1401, Servicebio) and imaged using either a Nikon fluorescence microscope or a Zeiss LSM 980 laser scanning confocal system.

### 4.12 Plasmids and transfection

The GEF‐H1‐Flag expression plasmid was generated by cloning the complete human GEF‐H1 coding sequence into the pCMV3‐C‐Flag expression vector. Site‐directed mutagenesis was utilized to incorporate individual amino acid replacements—namely Y394A, S399A, S324A, and C53R—as well as to generate domain‐truncation variants lacking the DH, PH, or zinc‐finger domains. In all constructs, an epitope tag (Flag) was attached to the C‐terminal end of GEF‐H1. Each plasmid was validated via Sanger sequencing, and transient transfection into the designated cell lines was performed with Lipofectamine 3000.

### 4.13 Infection of target cells by lentivirus

Healthy target cells were plated in a 24‐well culture plate at a concentration of 3 × 10^5^ cells per millilitre, with 500 µL per well, yielding approximately 1.5×10^5^ cells per well. The seeding density was adjusted slightly according to the cell growth rate to ensure 30%–50% confluency at the time of viral infection on the following day. The cells were cultured for approximately 12–16 h under standard conditions (37°C) under an atmosphere with controlled humidity and 5% carbon dioxide.

A half‐volume infection protocol was employed. Before infection, the viral stock was thawed slowly on ice. The initial culture medium was aspirated, and 1 mL of newly prepared medium—constituting half of the total 2 mL volume required per well in a six‐well plate—was then replenished. The required lentiviral volume was determined according to the desired multiplicity of infection (MOI), using the following calculation:

Lentivirus volume (µL) = (MOI × total cell count)/viral titer (TU/mL) × 1000 was added to the wells. For cell lines requiring polybrene to enhance transduction, an appropriate amount was added simultaneously. After 4 h of infection, the culture volume was replenished to 2 mL with complete medium.

At 10–24 h post‐infection, the original viral suspension was aspirated and replaced with fresh culture medium containing all required supplements. For viruses encoding a green fluorescent protein (GFP) reporter, transduction efficiency was preliminarily assessed by fluorescence microscopy at 48 h, with peak expression typically observed around 72 h. To select stably transduced cells, the culture medium was substituted with fresh complete medium that includes a precisely optimized concentration of puromycin—specifically calibrated for viral vectors carrying the puromycin resistance gene. The lentivirus was purchased from Corvus Biotechnology.

### 4.14 Patients

Before blood collection, documented consent was secured from all participants who took part in the research. Venous blood was drawn 24 h following admission to the surgical ICU. Serum was isolated via centrifugation at 3000×*g* for 25 min at 4°C and then divided into portions and kept at −80°C pending downstream assays. Healthy controls were carefully selected to exclude individuals with any history of autoimmune, chronic inflammatory, or active infectious conditions. A total of nine sepsis patients contributed blood samples—six for quantitative mRNA analysis and three for protein‐level assessment. All procedures adhered strictly to national ethical guidelines and to the ethical guidelines outlined in the Declaration of Helsinki and the Declaration of Istanbul.

### 4.15 PBMCs

Human peripheral blood mononuclear cells (PBMCs) were isolated from blood samples obtained from sepsis patients and healthy donors. The use of PBMCs was conducted in accordance with institutional ethics guidelines and approved protocols at Jinling Hospital. PBMCs were collected from both sepsis patients and HCs using density gradient centrifugation. Briefly, 4 mL of LymphoprepTM (07801, STEMCELL) was added to a 15‐mL SepMateTM tube (85415, STEMCELL). Blood samples (2 mL each) were diluted with an equal volume of PBS and carefully layered on top of the LymphoprepTM solution. The mixture was then centrifuged at 800 × g for 20 minutes at room temperature with the brake disengaged. Following centrifugation, the upper plasma layer and PBMC interface were collected and subjected to an additional centrifugation step at 800 × g for 3 minutes at room temperature for further processing and analysis.

### 4.16 Mass spectroscopy

First, the NCM460 cells that were co‐cultured with the LPS‐prepared NK92MI cells were washed with PBS solution, and then lysed to enable immunoprecipitation using a specific antibody targeting Gzma. The resulting immune complexes were resolved via sodium dodecyl sulphate–polyacrylamide gel electrophoresis (SDS–PAGE) and visualized using Coomassie Brilliant Blue staining. Following staining, the gel was rinsed twice and incubated at ambient temperature for 30 min to remove excess dye. Next, the gel pieces underwent dehydration and reduction steps before being subjected to overnight trypsin digestion (16 h, 37°C). Peptide fragments released from each excised band were extracted, pooled, and lyophilized to dryness. Synthetic peptides were dissolved in nano‐HPLC loading buffer and automatically injected onto a trapping column (100 µm × 20 mm, RP‐C18, Thermo Fisher Scientific). Chromatographic separation was performed using an analytical column (75 µm × 150 mm, RP‐C18, Thermo Fisher Scientific) with a flow velocity of 300nL/min, employing a 30 min linear gradient of mobile phase. Peptides recovered after elution were ionized online and analyzed by nanoelectrospray ionization collision‐induced dissociation mass spectrometry using an LTQ Orbitrap Velos Pro mass spectrometer (Thermo Fisher Scientific). Each LC–MS/MS run lasted 105 min and functioned in positive‐ion detection mode. Mass spectra were acquired with an electrospray voltage set to 1.8 kVand a capillary heated to 150°C. Before analysis, the instrument was calibrated using standard reference compounds. Full‐scan mass spectrometry (MS) data were acquired over an *m/z* range spanning from 350 to 1800. In data‐dependent acquisition mode, the 15 most intense precursor ions detected in each full‐scan MS spectrum were sequentially isolated and fragmented via CID. CID parameters included a collision energy setting normalized to 35%, an activation *q*‐value set at.25, and an activation duration of 30 ms. To prevent repeated selection of the same precursors, dynamic exclusion was enabled with a timeout period set to half the chromatographic peak width—specifically, 30 s. All MS spectra were recorded in profile format, whereas MS/MS spectra were acquired in centroid mode to optimize data storage efficiency. Subsequent database searching was carried out with Mascot software (version 2.3, Matrix Science Ltd., London, UK), yielding protein identifications with statistical confidence ≥95% based on scoring criteria.

### 4.17 Simulation of the interaction between Gzma and GEF‐H1

GRAMM is a rigid docking method employed for protein–protein docking. In this approach, the three‐dimensional structures of both the ligand and the receptor remain fixed throughout the docking process, and the geometric complementarity of the protein surfaces is utilized to identify potential binding sites. Beginning with the name of the target gene, we first retrieved the corresponding protein sequence from the UniProtKB database. This sequence was then submitted to SWISS‐MODEL for structure prediction, yielding the most reliable three‐dimensional structural model. The generated PDB file served as the input structure for interface analysis conducted using GRAMM. Following computational processing, a total of 10 docking conformations were generated, with the top‐ranked conformation exhibiting the most favourable binding characteristics. It is widely accepted that a binding free energy value below −4 kcal/mol indicates a potentially viable interaction, with lower values reflecting greater binding stability.

### 4.18 Paracellular transport of FITC‐dextran

Permeability across the intercellular space was assessed by measuring the translocation of fluorescein isothiocyanate (FITC)‐ fluorescently tagged dextran across epithelial monolayers cultured in Transwell inserts, following a protocol originally described by Balda et al.^45^ and subsequently refined by Michikawa et al.^46^ Specifically, FITC‐dextran was introduced into the basolateral compartment at a concentration of.1 mg/mL achieved at the end of the procedure. Following a 15 min equilibration period, samples were drawn from the upper chamber, and fluorescence signals were quantified on a Thermo Scientific Fluoroskan Ascent FL microplate fluorometer (Waltham, MA, USA), employing an excitation wavelength of 485 nm and monitoring emitted light at 538 nm. Quantification of FITC‐dextran concentration was derived from a standard curve correlating fluorescence signal to known concentrations. Apparent permeability was reported as a percentage of the theoretical saturation concentration—calculated as.26 mg FITC‐dextran dissolved in 4.1 mL total Transwell medium, yielding.0634 mg/mL. Data are reported as mean ± SEM. Non‐parametric statistical comparisons were performed using the Kruskal–Wallis nonparametric test. Subsequently, pairwise comparisons among multiple groups were conducted using Dunn's post hoc test. Statistical significance was defined as *p* < .01 (indicated by **).

### 4.19 Transepithelial resistance detection

Transepithelial resistance (TEER) detection was performed using a Millicell ERS‐2 volt‐ohmmeter in combination with an STX01 electrode. Human intestinal epithelial cells were seeded onto Transwell inserts featuring.4 µm pores and a surface area of.33 cm^2^, then cultured for three weeks to allow full differentiation into confluent monolayers; the culture medium was replenished every 48–72 h. Before TEER assessment, the standard growth medium was substituted with a serum‐free formulation. The electrodes were disinfected using 70% ethanol and subsequently washed thoroughly with phosphate‐buffered saline (PBS). The final TEER value (expressed in Ω·cm^2^) was obtained by first subtracting the background resistance contributed by cell‐free inserts from the total measured resistance, and then multiplying the resulting difference by the effective membrane surface area. A TEER value consistently exceeding 300 Ω·cm^2^ was required to confirm the formation of a fully integrated epithelial barrier. The experiment was carried out in triplicate under identical conditions, and the findings are presented as average values accompanied by the standard deviation. Statistical comparisons among groups were performed using single‐factor ANOVA with post hoc Tukey's HSD test, implemented in GraphPad Prism9. Statistical significance was defined as a *p*‐value below the conventional threshold of.05.

### 4.20 Statistical analysis

Data in the figure legends are presented as the mean value accompanied by the SD. Each experiment was independently repeated a minimum of three times, and reproducible findings were consistently obtained. Statistical analyses were carried out using GraphPad Prism 9. Normality of data distribution was evaluated via the Shapiro–Wilk test. When data met assumptions of normality and equal variance, comparisons between two independent groups were performed using a two‐tailed independent‐samples *t*‐test; where applicable—as noted in specific figures—the paired *t*‐test was adopted instead. For datasets violating normality, nonparametric alternatives were applied: the Mann–Whitney *U*‐test was employed to compare two independent groups, whereas the Kruskal–Wallis test was used for comparisons across three or more independent groups. Either one‐way or two‐way analysis of variance (ANOVA) was applied to multi‐group analyses, subsequently accompanied by suitable post hoc analyses when warranted. Correlation strength and direction were assessed using either Pearson's (for linear, normally distributed relationships) or Spearman's (for monotonic, non‐normal relationships) correlation coefficient, depending on data characteristics. Survival outcomes were estimated using the Kaplan–Meier method, and intergroup comparisons were conducted via the log‐rank test. The thresholds for statistical significance were defined as: *p < .05, **p < .01, ***p < .001, and ****p < .0001.

## AUTHOR CONTRIBUTIONS

Zexing Lin, Haiyang Jiang, Chujun Ni, and Yun Zhao conceptualized and planned the experimental framework. Experimental execution and subsequent data interpretation were carried out by Zexing Lin, Xuanheng Li, Chujun Ni, Runnan Wang, Yilong Yu, Weijie Li, and Bo Liao. Sequencing data analysis was conducted independently by Haiyang Jiang and Huan Yang. Research oversight and guidance were provided by Xiuwen Wu, Yun Zhao, and Jianan Ren. The initial manuscript draft was prepared by Zexing Lin, Haiyang Jiang, and Chujun Ni. Peizhao Liu undertook the computational prediction of protein structures. Compound screening efforts were supported by Haiyang Jiang and Weijie Li. Patient‐derived sample preparation was performed by Liting Deng, Jiaxin Yang, and Yue Chao. Critical revision of the submitted document was completed by Xiuwen Wu and Yun Zhao. All contributors examined the final version and granted their approval for submission.

## CONFLICT OF INTEREST STATEMENT

The authors declare no conflict of interest.

## ETHICS STATEMENT

All clinical studies adhered to the ethical standards outlined in the Declaration of Helsinki. The procurement of human blood specimens received formal ethical approval from the Institutional Review Board (IRB) of Jinling Hospital (Approval No. 2020DZGZRZX‐065). All participants, or their legally appointed representatives where required, provided written consent before involvement in the study. Animal A series of empirical tests was carried out strictly in line with national and institutional guidelines for the Well‐being of Animals and were reviewed and approved by the Institutional Animal Care and Use Committee (IACUC) of Nanjing Medical University (Protocol No. IACUC‐2508016).

## Supporting information



Supporting Information

## Data Availability

scRNA‐seq data: Transcriptomic profiles. These datasets have been archived in the NCBI SRA under accession IDs SRR18159260–SRR18159271.
