# Supplementary material for: Mechanism of Gzma‐mediated GEF‐H1 activation in intestinal epithelial cells leading to intestinal barrier dysfunction in sepsis
Source: Clin Transl Med. 2026 Apr 6;16(4):e70651. doi: 10.1002/ctm2.70651 (PMC13053666; doi:10.1002/ctm2.70651)

### Figure S1: Elevated Expression of Gzma in Sepsis and lts Mediation of Intestinal Epithelial Barrier Dysfunction

(A) Violin plot showing Gzma gene expression in PBMCs from HCs and sepsis patients (n = 393, transcriptomic data by Arjun Baghela et al.). (B) HE staining of ileum, lung, and liver tissues from WT and CLP mice (scale bar = 100 μm). (C) RT-qPCR analysis of Occludin, Claudin1, ZO-1 and E-cadherin in intestinal tissues from CLP-induced sepsis model mice and WT sham controls. Data: mean ± SEM; n = 3; **p < 0.01, Student’s t-test. (D) Immunofluorescence staining of ZO-1 (red, top) and E-cadherin (green, bottom) in intestinal tissues; nuclei stained with DAPI (blue); scale bar = 20 μm. (E) IL-1β and TNF-α mRNA levels in PBMC of sepsis patients positively correlated with SOFA score (r=0.6655, 0.6667; all P<0.01). (F) Western blot of Occludin, Claudin1, ZO-1, and E-cadherin in HT-29 cells after co-culture with LPS-pretreated NK92MI cells (β-actin loading control). (G) TEER measurements in HT-29 cells post-co-culture; mean ± SEM; n = 3; ***p < 0.001 (one-way ANOVA). (H) FITC-dextran permeability assay in HT-29 cells post-co-culture; mean ± SEM; n = 3; **p < 0.01 (one-way ANOVA). (I) Immunofluorescence of E-cadherin in Caco2 cells (top: control; middle: NK92MI co-culture; bottom: LPS-stimulated NK92MI co-culture; green: E-cadherin; blue: DAPI; scale bar = 20 μm). (J) Western blot of Occludin, Claudin1, ZO-1, and E-cadherin in NCM460, HT-29, and Caco2 cells after Gzma electroporation (β-actin loading control). (K) RT-qPCR analysis of these genes in the same cell lines after Gzma electroporation.

### Figure S2: Activation of the GEF-H1/RhoA Signaling Axis by Gzma under Sepsis Conditions

(A) GO enrichment analysis of transcriptome sequencing results following the co-culture of human colonic epithelial cells (NCM460) with LPS-pretreated NK92MI cells. The enriched gene pathways include "cytoskeleton organization" and "microtubule dynamics"; the color scale represents the level of enrichment significance. (B) RT-qPCR analysis of cytoskeleton-related gene expression in NCM460 cells after co-culture with LPS-pretreated NK92MI cells (mean ± SEM, n = 3 per group; ns > 0.05, *p < 0.05, **p < 0.01, ***p < 0.001, ****p < 0.0001, Student's t-test). (C) Immunofluorescence staining of β-tubulin (red) in Caco2 cells following co-culture with LPS-pretreated NK92MI cells. Nuclei were stained with DAPI (blue); top row: control group; bottom row: co-culture group; scale bar = 20 μm. (D) RhoA G-LISA activity assay in HT-29 cells after co-culture with LPS-pretreated NK92MI cells (mean ± SEM, n = 3 per group; ***p < 0.001, ****p < 0.0001, one-way ANOVA). (E) Western blot analysis of p-GEF-H1, GEF-H1, p-MLC2, MLC2, p-LIMK, LIMK, p-Cofilin, Cofilin, and β-actin in HT-29 cells after co-culture with LPS-pretreated NK92MI cells; β-actin was used as a loading control. (F) RhoA G-LISA activity assay in three human intestinal epithelial cell lines (NCM460, HT-29, and Caco2) following electroporation with Gzma (mean ± SEM, n = 3 per group; ***p < 0.001, ****p < 0.0001, one-way ANOVA). (G) Western blot analysis of p-GEF-H1, GEF-H1, p-MLC2, MLC2, p-LIMK, LIMK, p-Cofilin, Cofilin, and β-actin in three human intestinal epithelial cell lines following electroporation with Gzma; β-actin was used as a loading control.

### Figure S3: Knockout or Activation of GEF-H1 Regulates Transcriptional Levels of Tight Junction Proteins in CLP Mice

(A) Western blot analysis was performed to examine the expression of GEF-H1 protein in the intestines of WT and GEF-H1^⁻/⁻^ mice. β-Actin was used as the loading control sample. (B) RT-qPCR analysis of Occludin, Claudin1, ZO-1, and E-cadherin in ileal tissues from WT and GEF-H1^⁻/⁻^ mice following either sham operation or CLP treatment (mean ± SEM, n = 3/group; *p < 0.05, **p < 0.01, two-way ANOVA). (C) RT-qPCR analysis of Occludin, Claudin1, ZO-1, and E-cadherin in ileal tissues from WT mice subjected to sham operation, CLP treatment, GEF-H1 agonist Plinabulin treatment, or CLP combined with GEF-H1 agonist Plinabulin treatment (mean ± SEM, n = 3/group; *p < 0.05, two-way ANOVA).

### Figure S4: Knockout of GEF-H1 Protects Against Sepsis-Associated Epithelial Barrier Damage In Vitro

(A) Western blot analysis of WT PIEC cells and GEF-H1^-/-^ PIEC cells following co-culture with LPS-stimulated NK92MI cells. Target proteins included p-GEF-H1, GEF-H1, p-MLC2, MLC2, p-LIMK, LIMK, p-Cofilin, Cofilin, and β-actin (loading control). (B) Western blot analysis of WT PIEC cells and GEF-H1^-/-^ PIEC cells after co-culture with LPS-stimulated NK92MI cells. Detected proteins included Occludin, Claudin-1, ZO-1, E-cadherin, Gzma, and β-actin (loading control). (C)TEER assay to evaluate epithelial barrier function in WT and GEF-H1^-/-^ PIEC cells following co-culture with LPS-pretreated NK92MI cells (mean ± SEM, n = 3 per group; *p < 0.05, two-way ANOVA). (D) FITC-dextran permeability assay to assess paracellular permeability in WT and GEF-H1^-/-^ PIEC cells after co-culture with LPS-pretreated NK92MI cells (mean ± SEM, n = 3 per group; *p < 0.05, two-way ANOVA). (E) RT-qPCR analysis of mRNA expression levels of Occludin, Claudin-1, ZO-1 and E-cadherin in experimental groups (mean ± SEM, n = 3 per group; *p < 0.05, two-way ANOVA). (F) Western blot analysis of NCM460 cells transfected with Tet-on GEF-H1, induced for 0, 8, or 16 hours, and subsequently co-cultured with LPS-pretreated NK92MI cells. Detected proteins included p-GEF-H1, GEF-H1, p-MLC2, MLC2, p-LIMK, LIMK, p-Cofilin, Cofilin, and β-actin (loading control). (G) Western blot analysis of NCM460 cells transfected with Tet-on GEF-H1 (induced for 0, 8, or 16 hours) and co-cultured with LPS-pretreated NK92MI cells. Target proteins included Occludin, Claudin-1, ZO-1, E-cadherin, Gzma, and β-actin (loading control). (H) TEER assay to evaluate epithelial barrier function in NCM460 monolayers transfected with Tet-on GEF-H1 (induced for 0, 8, or 16 hours) and co-cultured with LPS-stimulated NK92MI cells (mean ± SEM, n = 3 per group; *p < 0.05, two-way ANOVA). (I) FITC-dextran permeability assay to assess paracellular permeability in NCM460 monolayers under the same treatment conditions as (C) (mean ± SEM, n = 3 per group; *p < 0.05, two-way ANOVA). (J) qRT-PCR quantification of mRNA expression levels of Occludin, Claudin-1, ZO-1, and E-cadherin in NCM460 cells after co-culture with LPS-stimulated NK92MI cells (mean ± SEM, n = 3 per group; *p < 0.05, **p < 0.01, ***p < 0.001, two-way ANOVA). (K) Immunofluorescence staining of ZO-1 (red), GEF-H1 (green), and DAPI (blue) in Caco-2 cells under the same treatment conditions as (F).

### Figure S5: Knockdown of GEF-H1 Protects Against Sepsis-Associated Epithelial Barrier Damage In Vitro

(A) Western blot analysis of GEF-H1 and β-actin expression (top panel) and corresponding quantitative analysis of GEF-H1 protein band intensity (bottom panel) in the human colonic epithelial cell line NCM460 following transfection with three distinct GEF-H1 siRNAs (siRNA1, siRNA2, siRNA3) or negative control (NC) siRNA. (B) Western blot analysis of Occludin, Claudin1, ZO-1, E-cadherin, and Gzma in three human intestinal epithelial cell lines (NCM460, HT-29, Caco2) after GEF-H1 knockdown (via siRNA1 or siRNA3) and subsequent co-culture with LPS-pretreated NK92MI cells (β-actin served as the loading control). (C) RT-qPCR analysis of mRNA expression levels of Occludin, Claudin1, ZO-1 and E-cadherin in NCM460 cells following GEF-H1 knockdown and co-culture with LPS-pretreated NK92MI cells (data presented as mean ± SEM, n = 3/group; ***p < 0.0001, two-way ANOVA). (D) Immunofluorescence staining of E-cadherin and ZO-1 in Caco2 cells after GEF-H1 knockdown and co-culture with LPS-pretreated NK92MI cells (green: E-cadherin; blue: DAPI; scale bar = 50 μm or 100 μm as indicated). (E) TEER measurements in NCM460, HT-29, and Caco2 cells following GEF-H1 knockdown and co-culture with LPS-pretreated NK92MI cells (data presented as mean ± SEM, n = 3/group; *p < 0.05, **p < 0.01, ***p < 0.001, two-way ANOVA). (F) FITC-dextran permeability assay in NCM460, HT-29, and Caco2 cells after GEF-H1 knockdown and co-culture with LPS-pretreated NK92MI cells (data presented as mean ± SEM, n = 3/group; *p < 0.05, **p < 0.01, ***p < 0.001, two-way ANOVA). (G) RhoA activity assessment via G-LISA assay in three human intestinal epithelial cell lines (NCM460, HT-29, Caco2) following GEF-H1 knockdown and co-culture with LPS-pretreated NK92MI cells (data presented as mean ± SEM, n = 3/group; ***p < 0.001, ****p < 0.0001, two-way ANOVA). (H) Western blot analysis of RhoA/ROCK signaling pathway-related molecules (GEF-H1, p-MLC2, MLC2, p-LIMK, LIMK, p-Cofilin, Cofilin) and Gzma in three human intestinal epithelial cell lines (NCM460, HT-29, Caco2) after GEF-H1 knockdown (via siRNA1 or siRNA3) and co-culture with LPS-pretreated NK92MI cells (β-actin served as the loading control).

### Figure S6: Overexpression of GEF-H1 Disrupts Epithelial Barrier Integrity in an In Vitro Model of Sepsis

(A) Western blot analysis of Occludin, Claudin1, ZO-1, E-cadherin, and Gzma in HT-29 overexpressing GEF-H1 following co-culture with LPS-pretreated NK92MI cells (β-actin was used as the loading control). (B) RT-qPCR analysis of mRNA expression levels of Occludin, Claudin1, ZO-1 and E-cadherin in NCM460 cells overexpressing GEF-H1 after co-culture with LPS-pretreated NK92MI cells (mean ± SEM, n = 3/group; *p < 0.05, **p < 0.01, ***p < 0.001, ****p < 0.0001, one-way ANOVA). (C) Immunofluorescence staining of E-cadherin in Caco2 cells overexpressing GEF-H1 following co-culture with LPS-pretreated NK92MI cells (green: E-cadherin; blue: DAPI nuclear staining; scale bar = 20 μm). (D) TEER was measured in HT-29 cells overexpressing GEF-H1 after co-culture with LPS-pretreated NK92MI cells (mean ± SEM, n = 3/group; *p < 0.05, ***p < 0.001, one-way ANOVA). (E) FITC-dextran was performed in HT-29 cells overexpressing GEF-H1 after co-culture with LPS-pretreated NK92MI cells (mean ± SEM, n = 3/group; *p < 0.05, ***p < 0.001, one-way ANOVA). (F) RhoA G-LISA activity assay in three human intestinal epithelial cell lines (NCM460, HT-29, Caco2) overexpressing GEF-H1 after co-culture with LPS-pretreated NK92MI cells (mean ± SEM, n = 3/group; **p < 0.01, ***p < 0.001, ****p < 0.0001, one-way ANOVA). (G) Western blot analysis of key molecules in the RhoA/ROCK signaling pathway (p-GEF-H1, p-MLC2, p-LIMK, p-Cofilin) and β-actin expression in three human intestinal epithelial cell lines overexpressing GEF-H1 after co-culture with LPS-pretreated NK92MI cells (β-actin served as the loading control).

### Figure S7: Epothilone A Attenuates Sepsis-Induced Intestinal Barrier Injury by Inhibiting GEF-H1 Activity via Phosphorylation

(A) NCM460 cells were treated with Epothilone A, Epothilone B, or Ansamitocin P-3 and co-cultured with LPS-pretreated NK92MI cells. Western blot analysis assessed p-GEF-H1, GEF-H1, and β-actin (loading control). (B) HT-29 cells were treated with 10 nmol DMSO, Epothilone A, Epothilone B, or Ansamitocin P-3, then co-cultured with LPS-pretreated NK92MI cells. Western blot was used to detect Occludin, Claudin1, ZO-1, E-cadherin, and Gzma, with β-actin as loading control. (C) NCM460 cells treated with 10 nmol of the same compounds were co-cultured and analyzed for mRNA levels of Occludin, Claudin1, ZO-1, and E-cadherin by RT-qPCR (mean ± SEM, n=3; *p < 0.05, **p < 0.01, one-way ANOVA). (D) NCM460, HT-29, and Caco2 cells were treated with 10 nmol of each compound and co-cultured. TEER was measured to evaluate barrier integrity (mean ± SEM, n=3; *p < 0.05, **p < 0.01, one-way ANOVA). (E) The same cell lines were tested in a FITC-dextran assay post-co-culture to assess paracellular permeability (mean ± SEM, n=3; *p < 0.05, **p < 0.01, ***p < 0.001, one-way ANOVA). (F) Caco2 cells were treated and co-cultured as above. Immunofluorescence staining visualized E-cadherin (green) and nuclei (blue, DAPI; scale bar = 20 μm). (G) RhoA activity in the three cell lines was analyzed using a G-LISA assay after treatment and co-culture (mean ± SEM, n=3; *p < 0.05, **p < 0.01, ***p < 0.001, one-way ANOVA). (H) Western blot analysis of RhoA/ROCK signaling (p-GEF-H1, p-MLC2, p-LIMK, p-Cofilin) in three human intestinal epithelial cell lines. Cells were treated with Epothilone A, Epothilone B, or Ansamitocin P-3 and co-cultured with LPS-pretreated NK92MI cells. β-actin was used as the loading control.

## Figure

### Figure 1


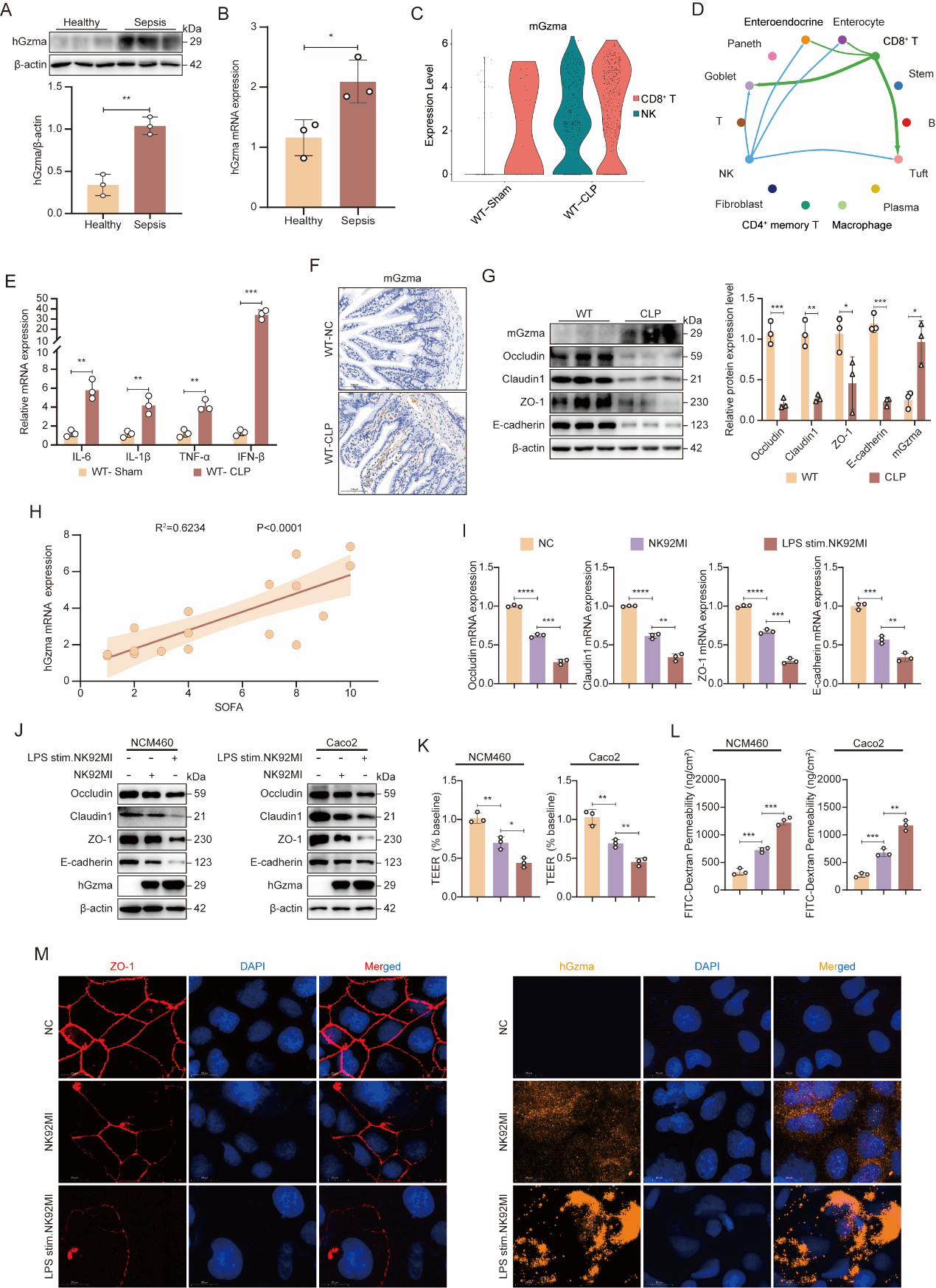


### Figure 2


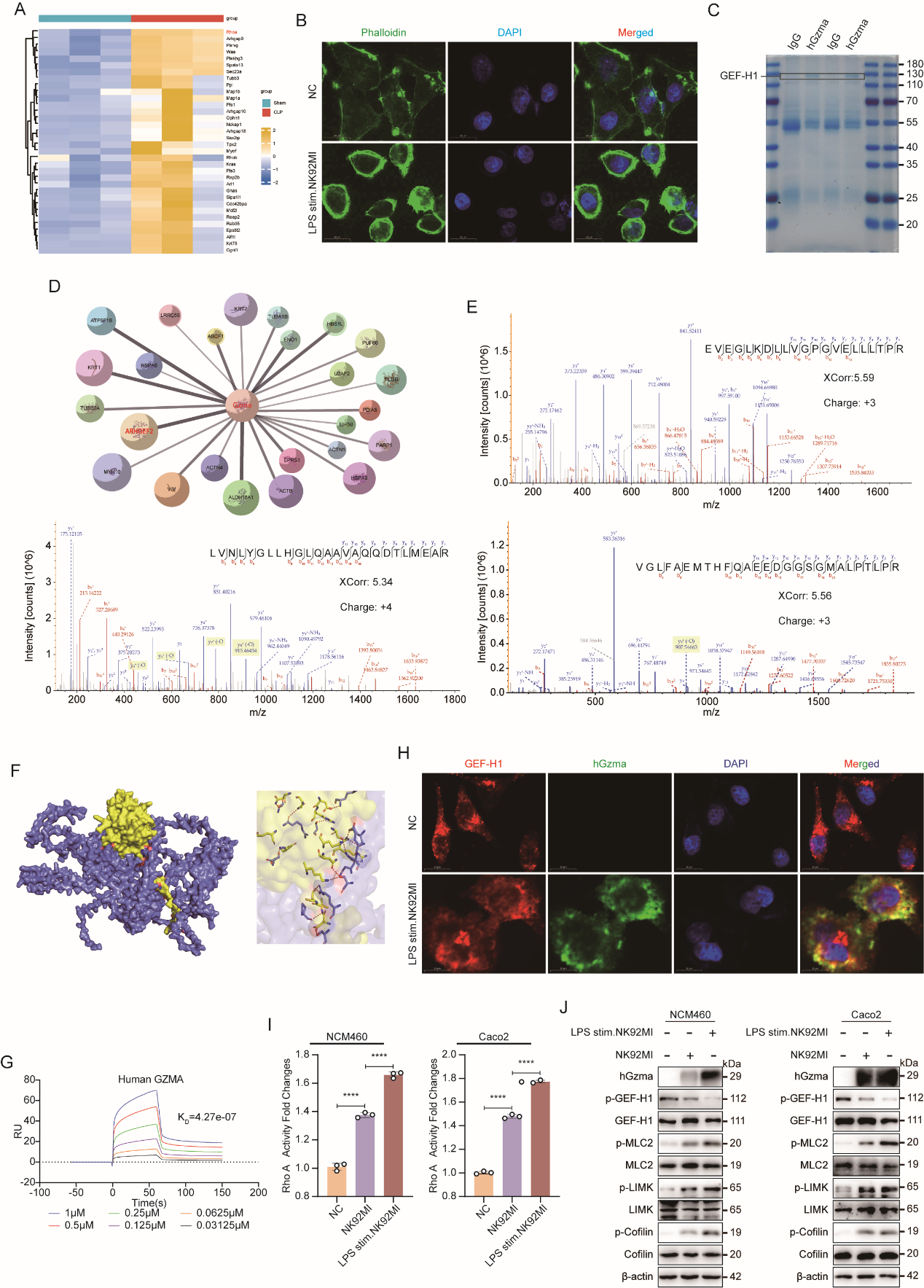


### Figure 3


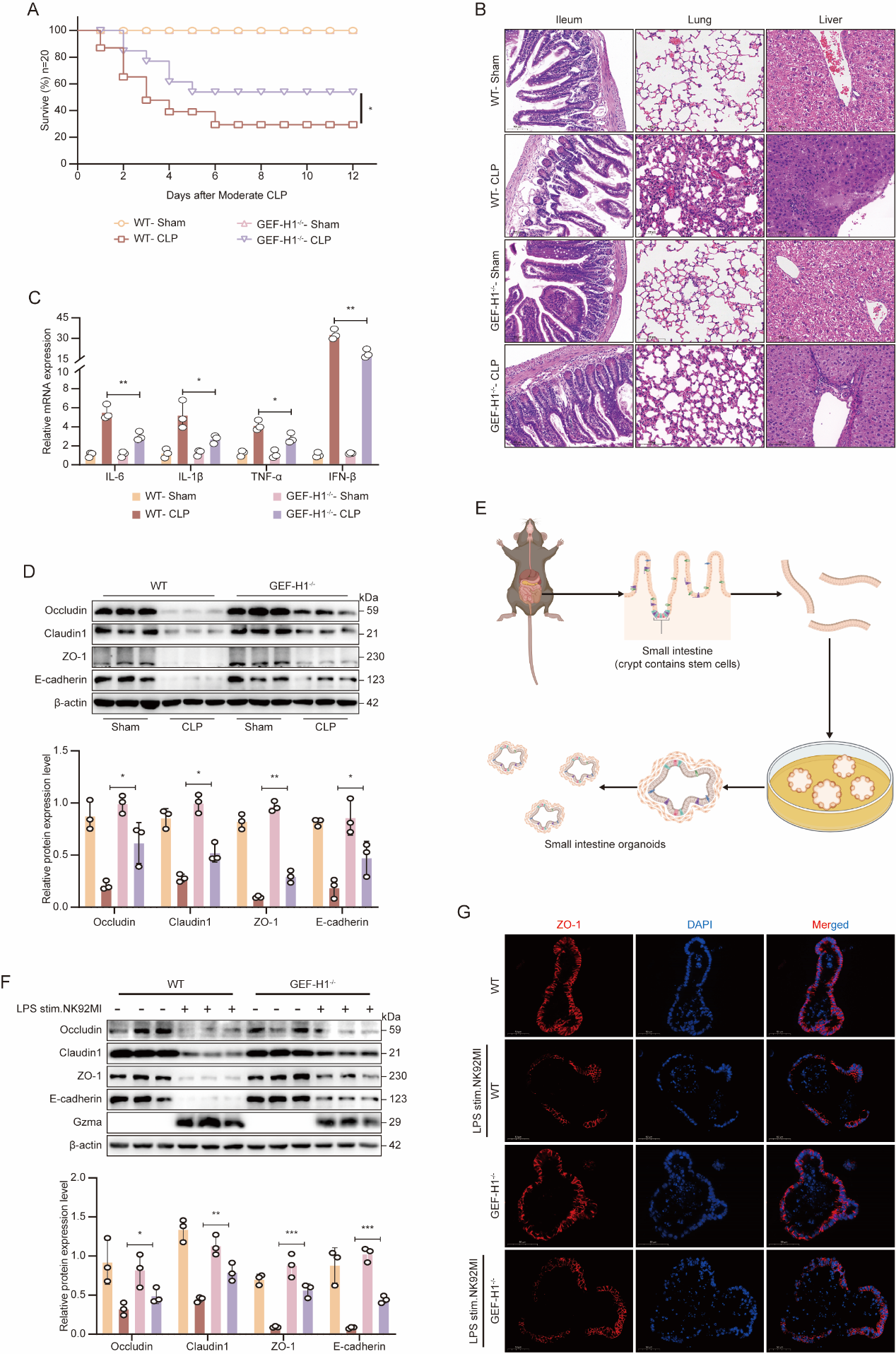


### Figure 4


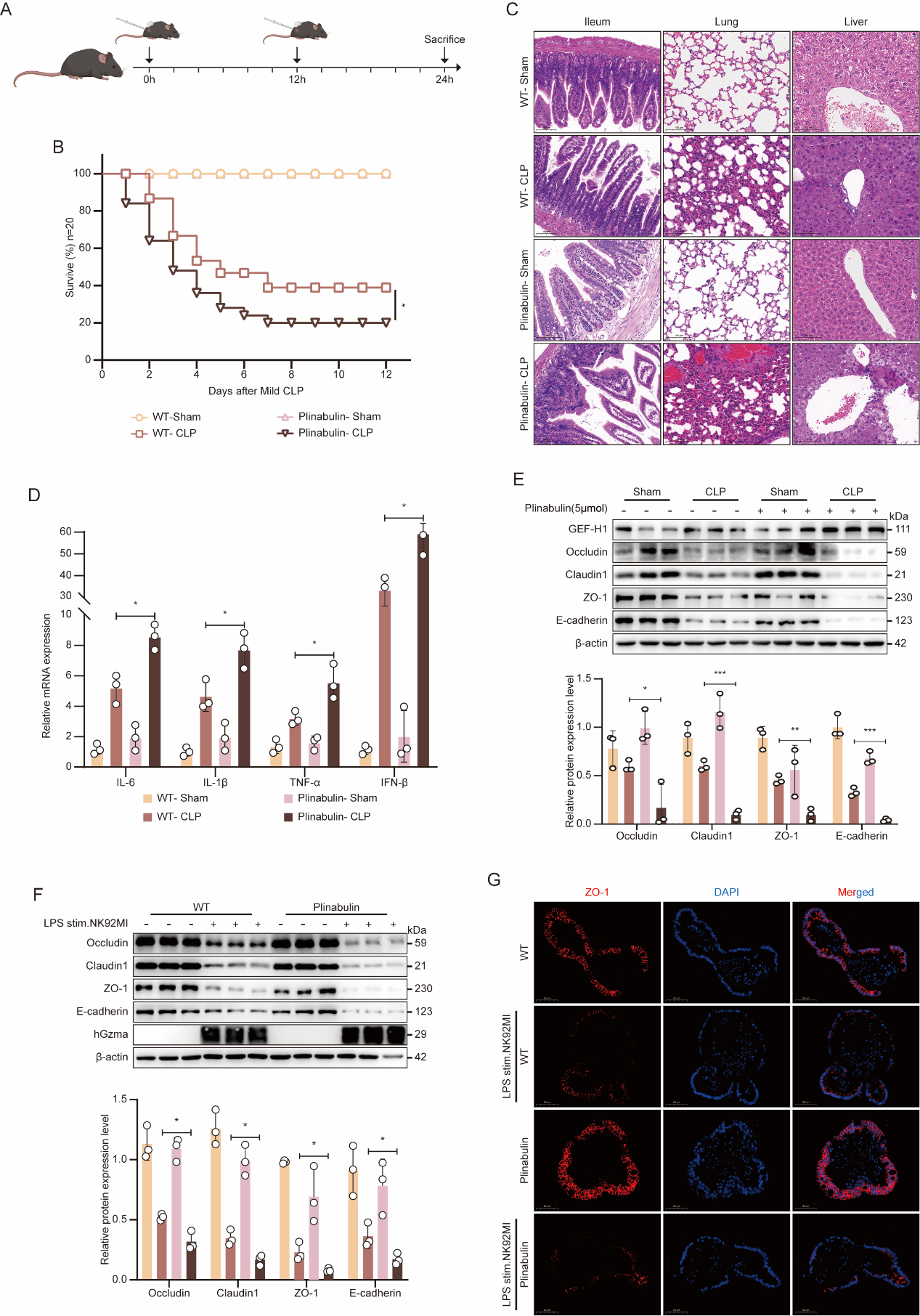


### Figure 5


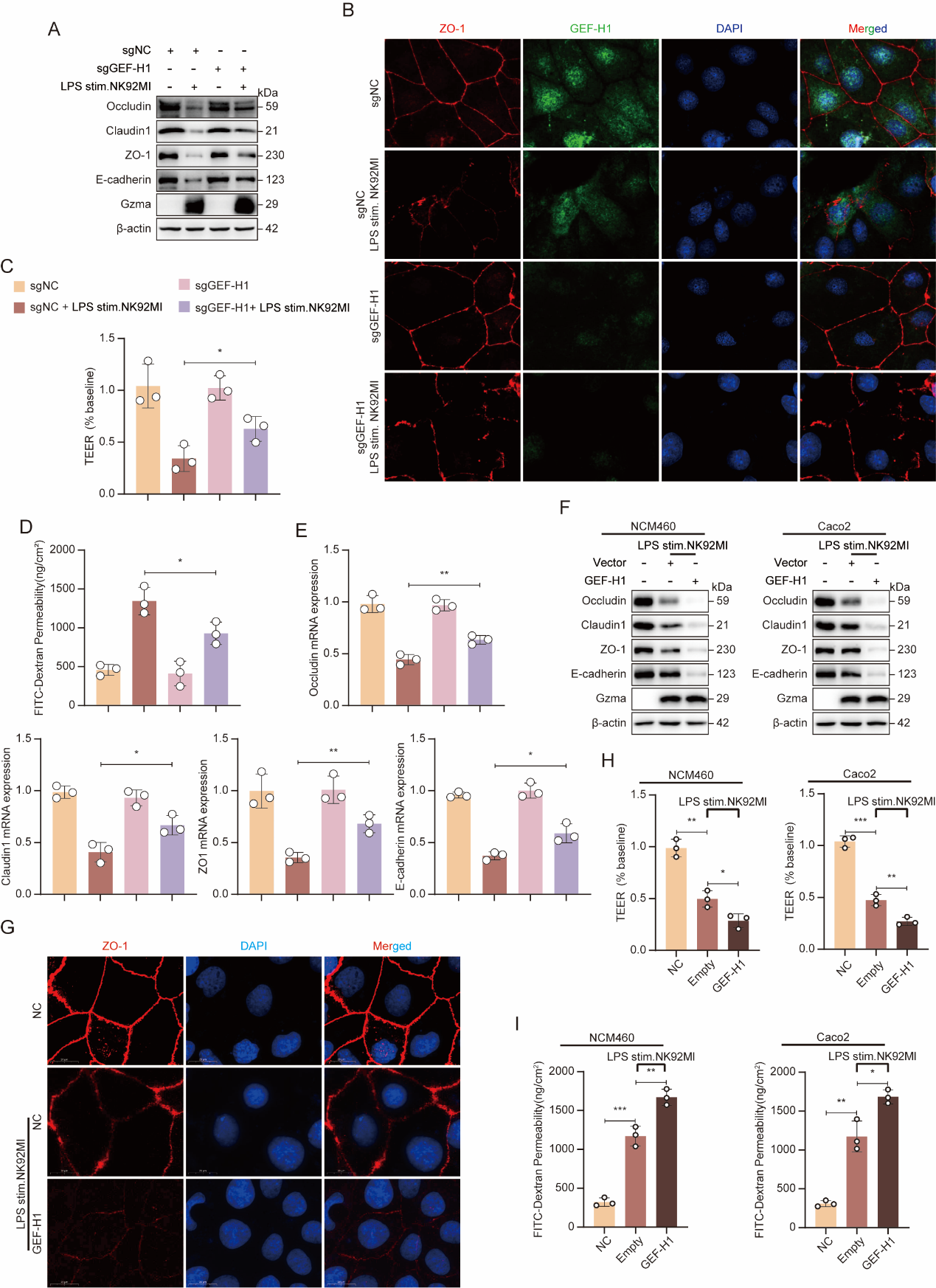


### Figure 6


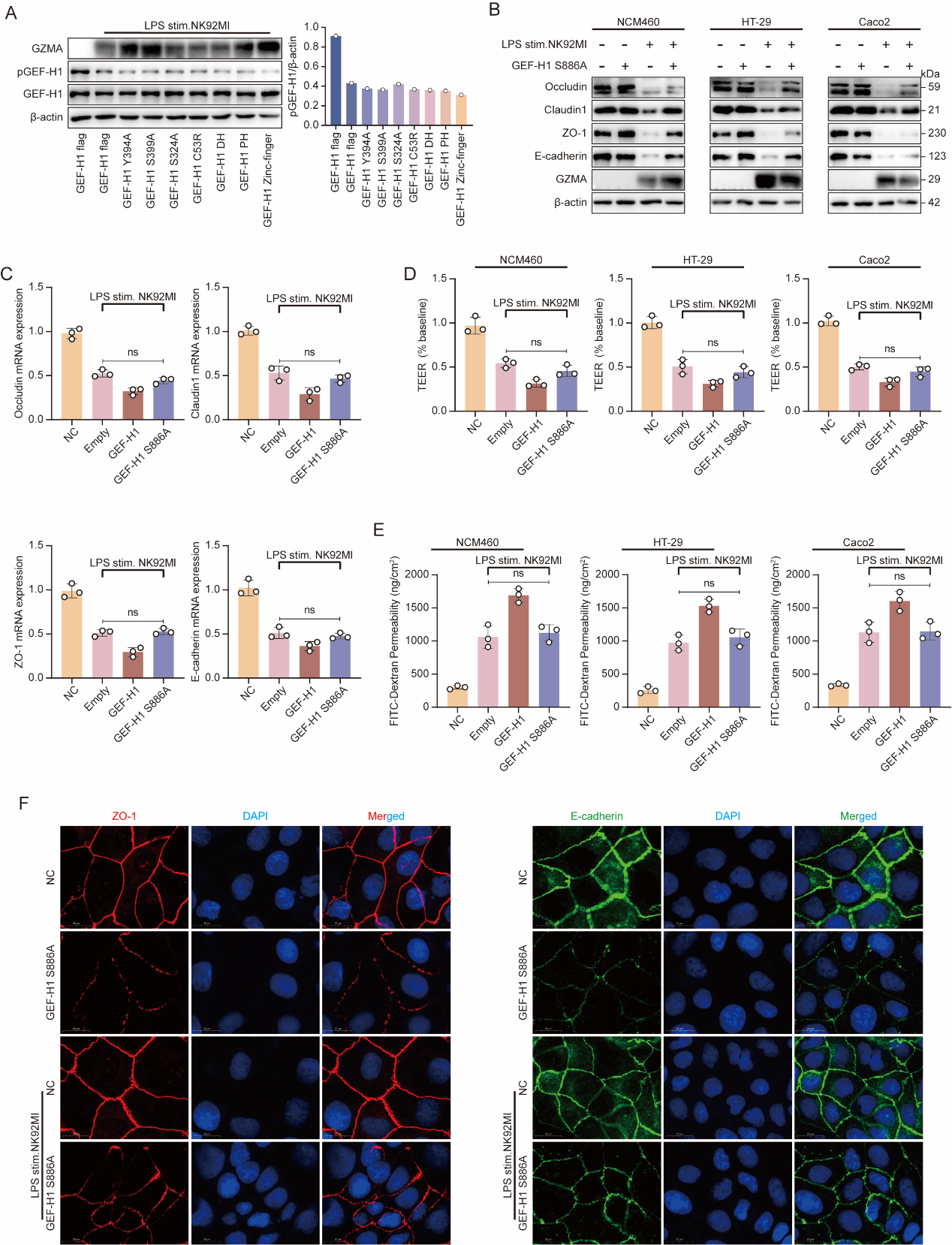


### Figure 7


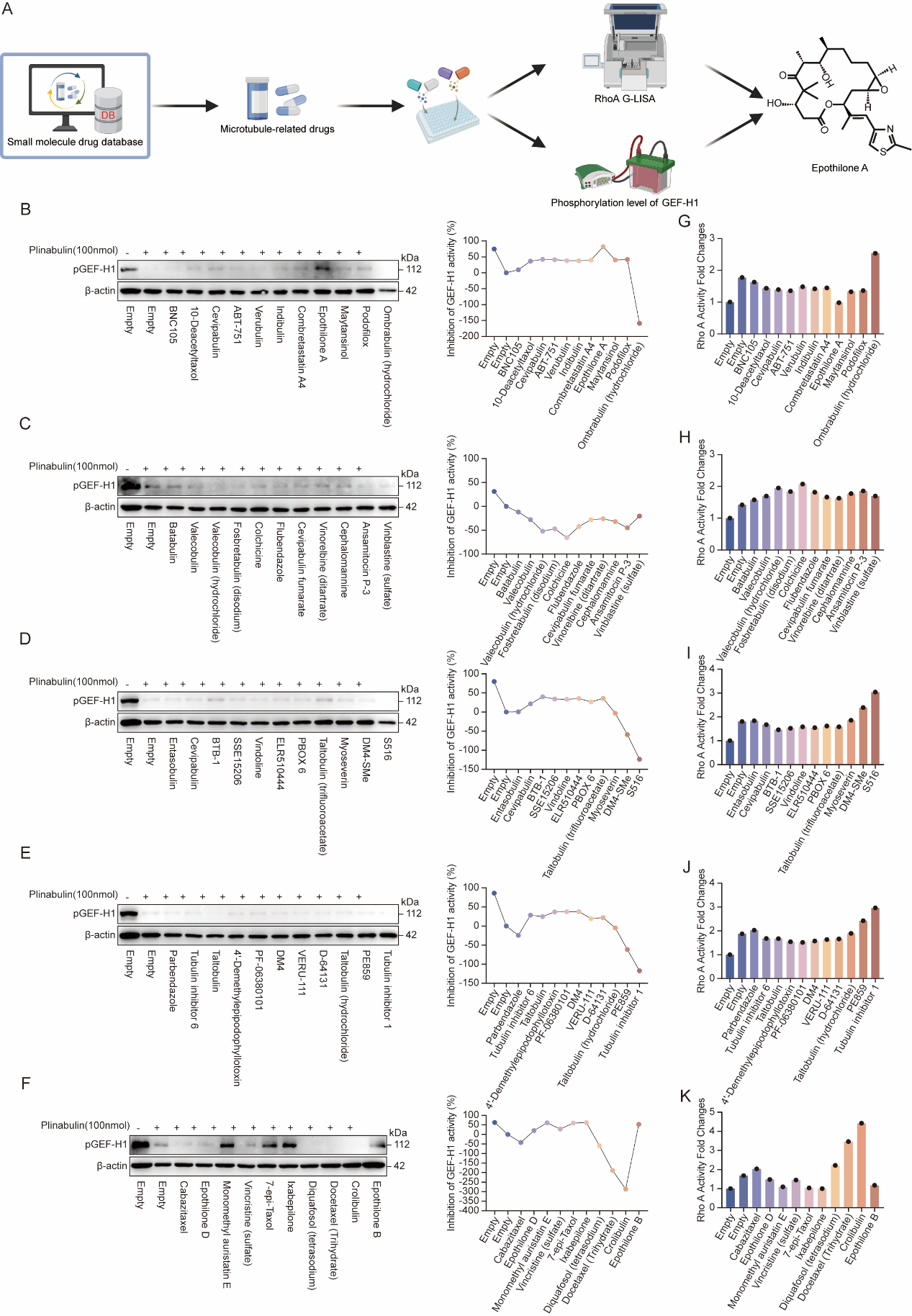


### Figure 8


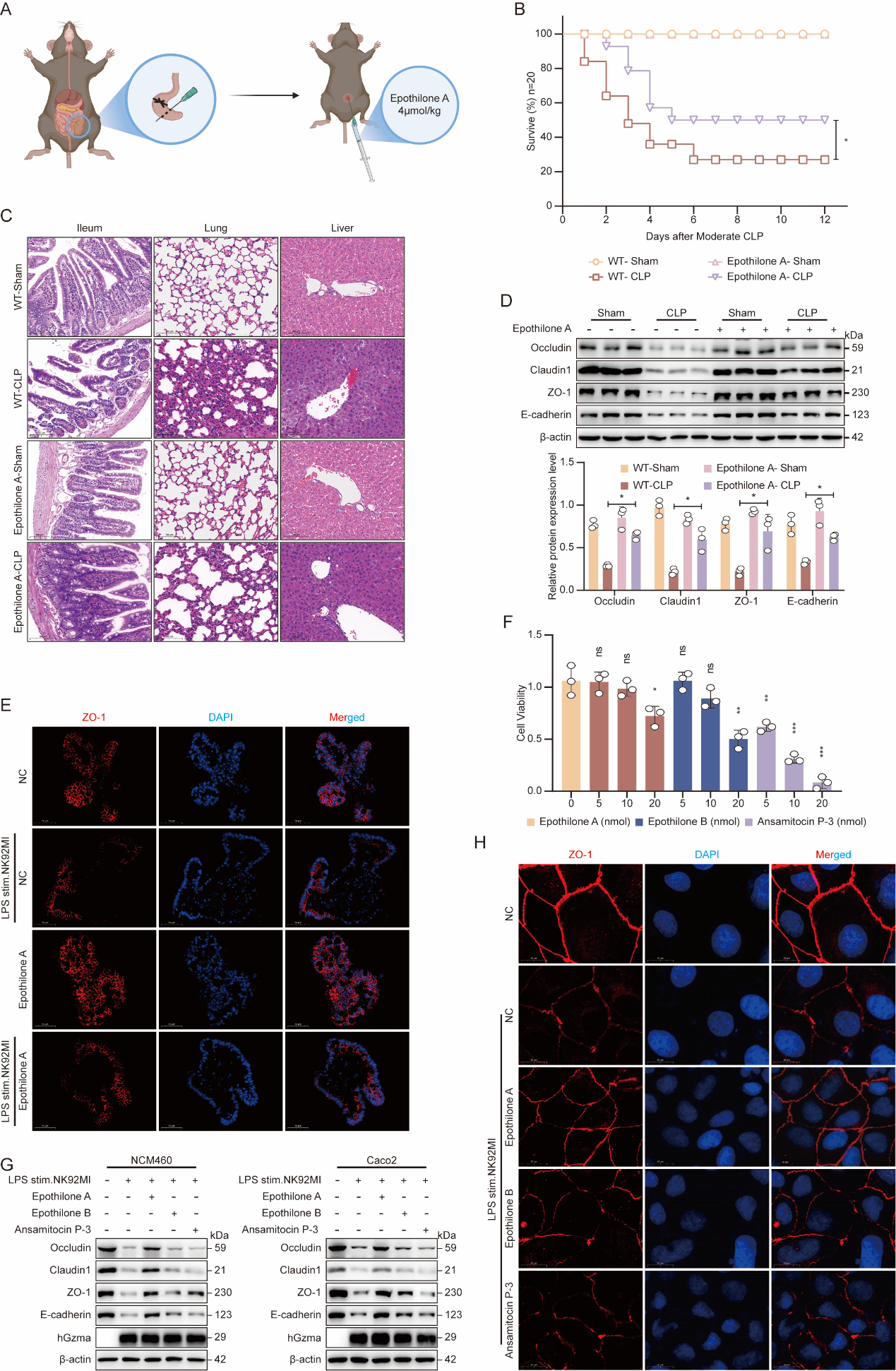


### Figure 9


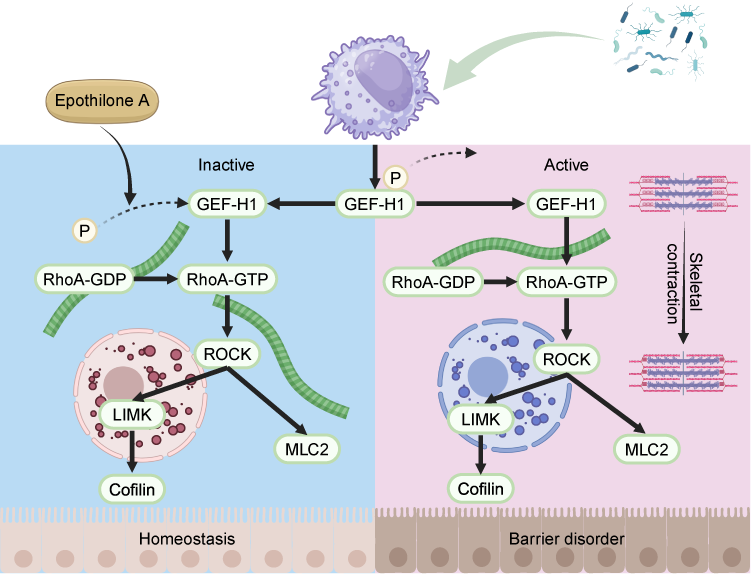


### Figure S1


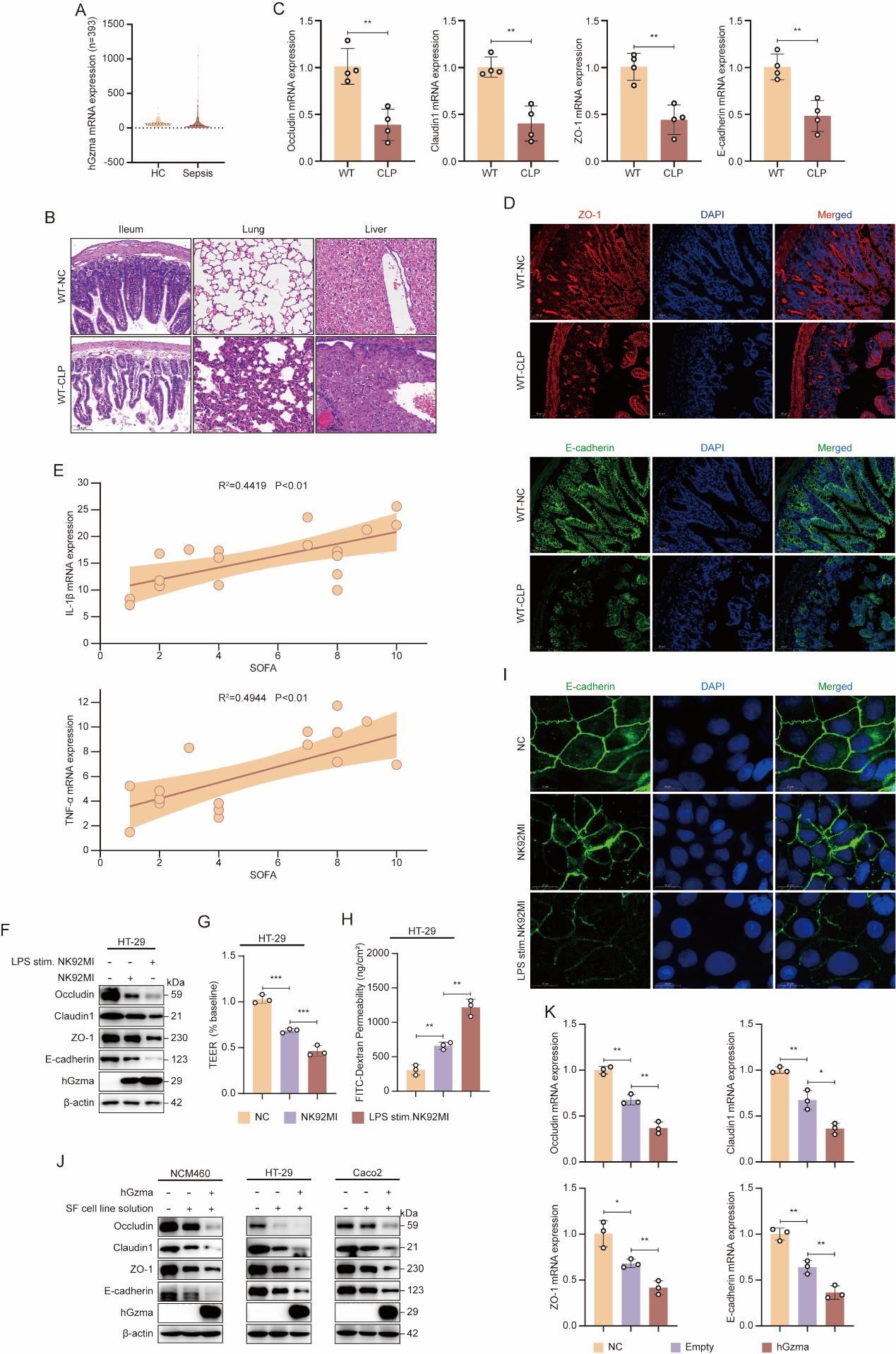


### Figure S2


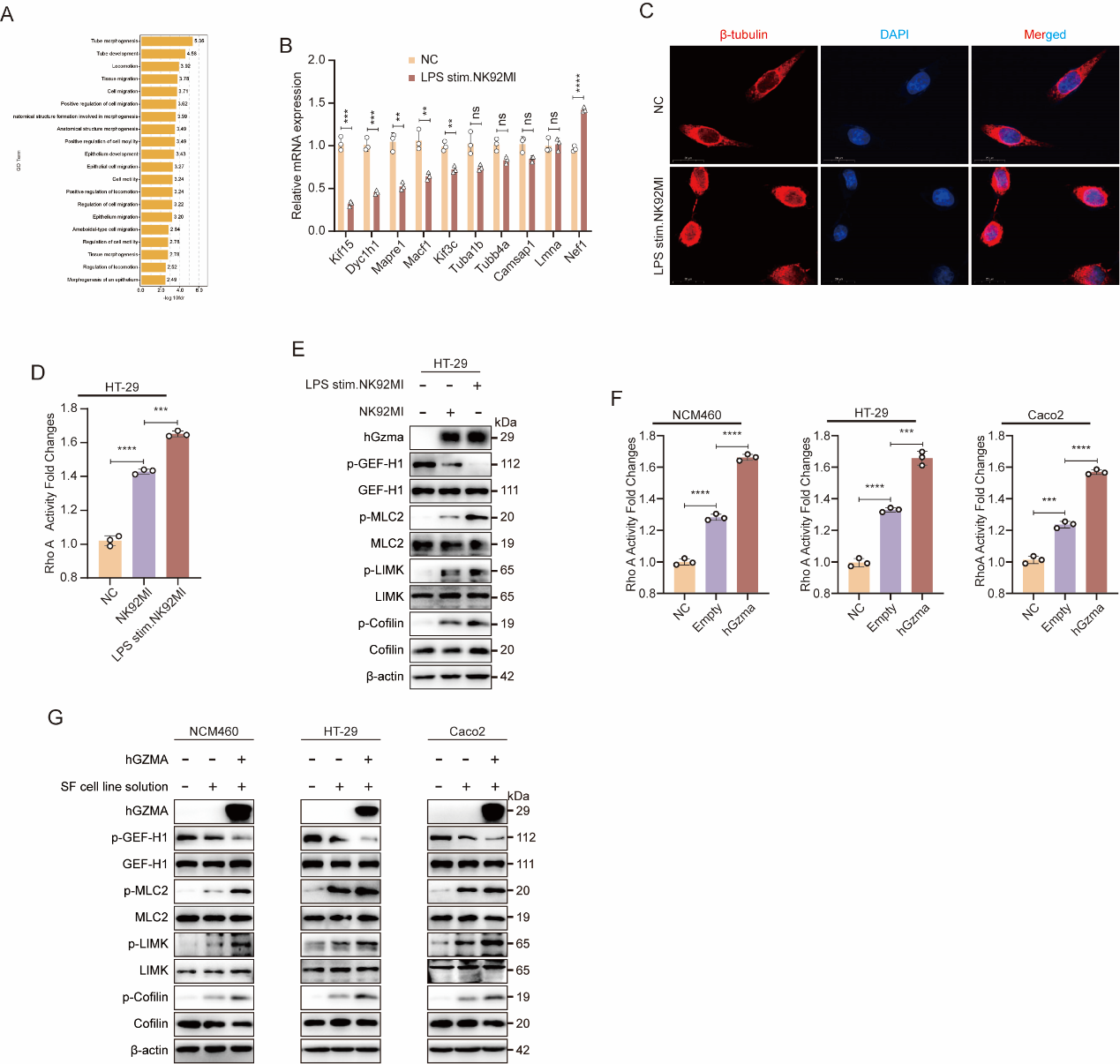


### Figure S3


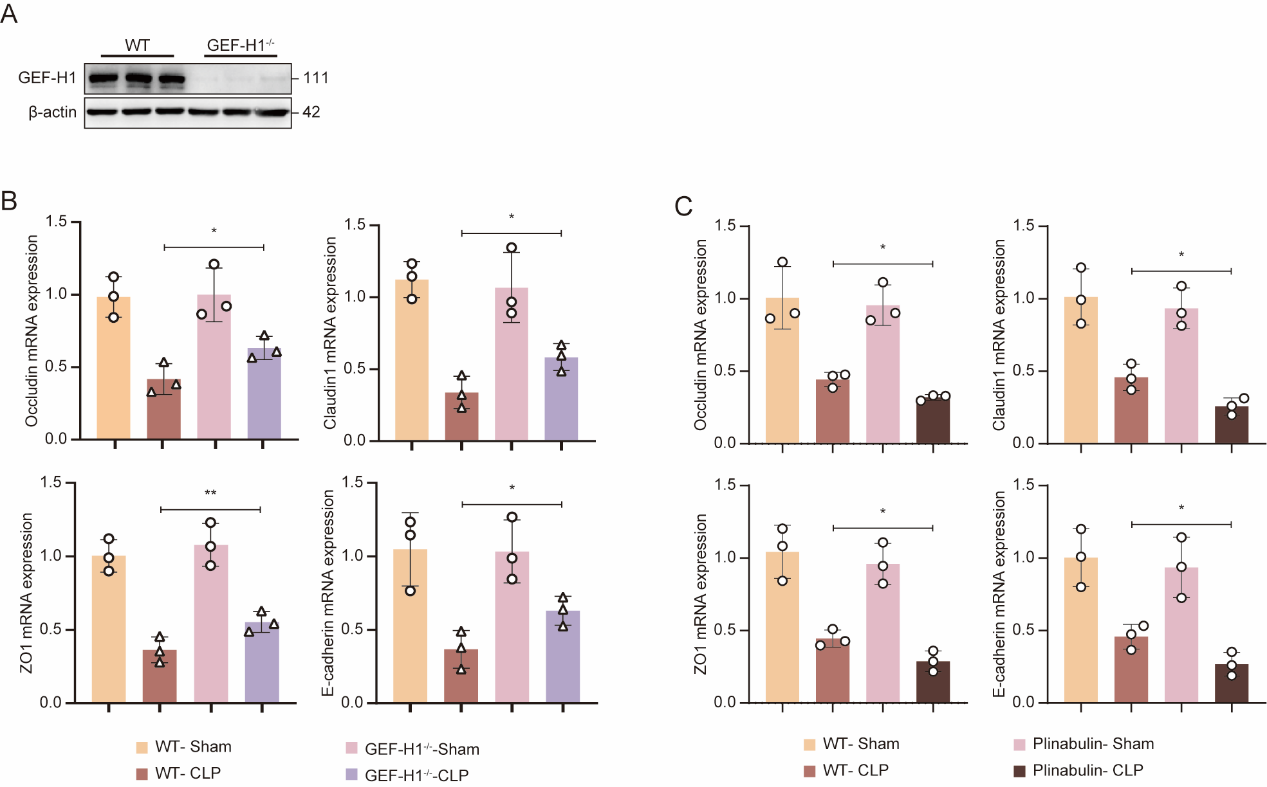


### Figure S4


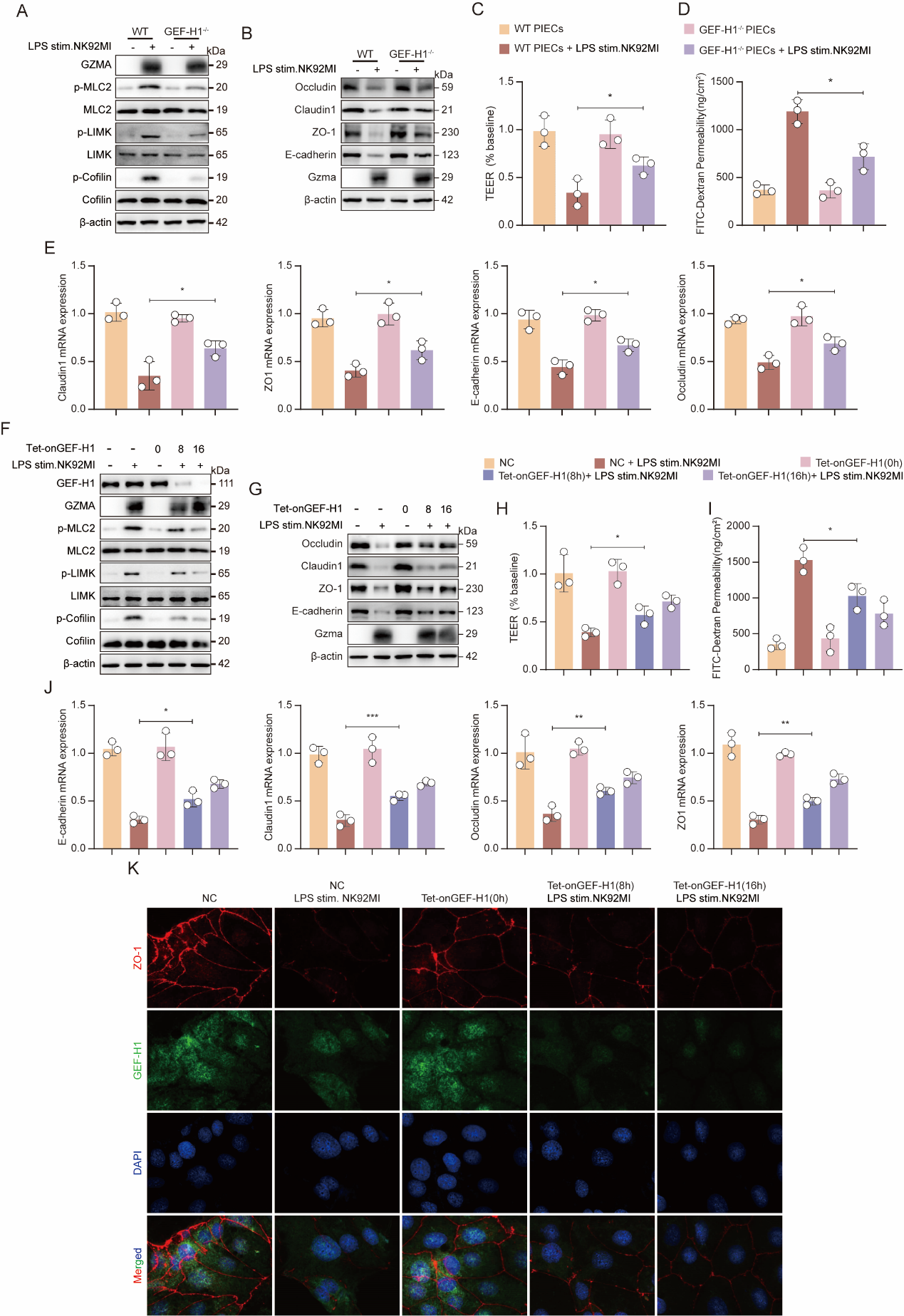


### Figure S5


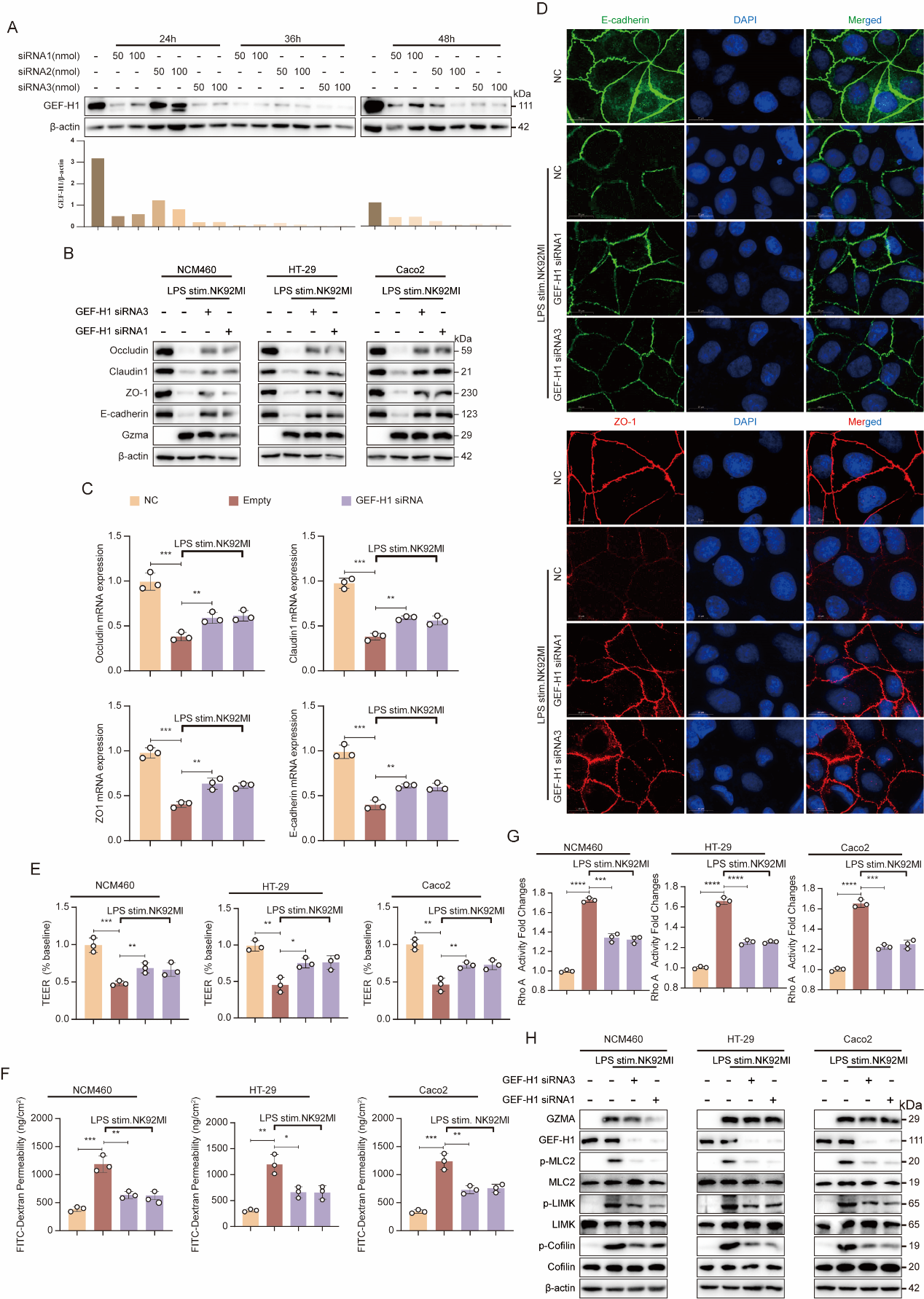


### Figure S6


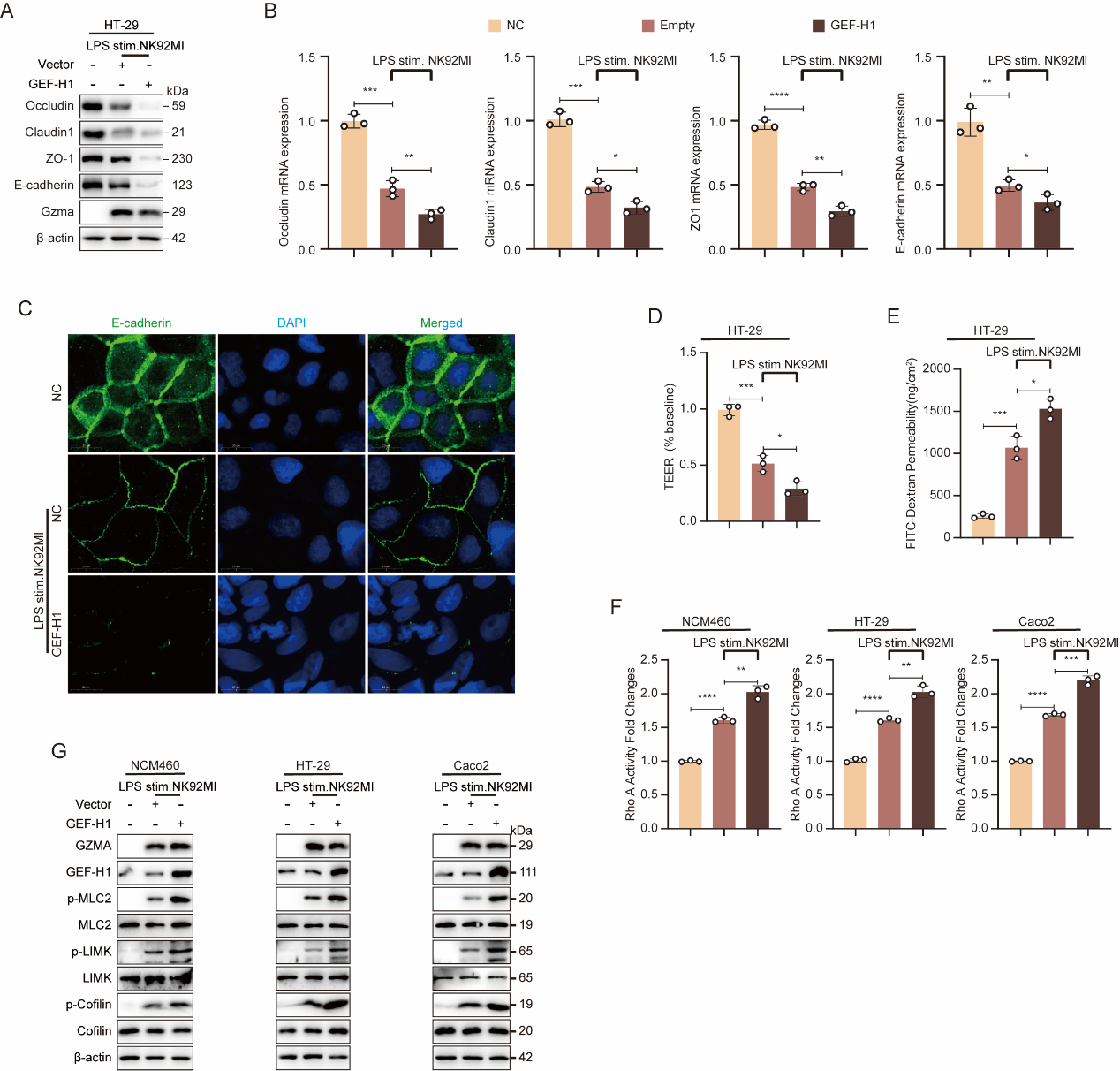


### Figure S7


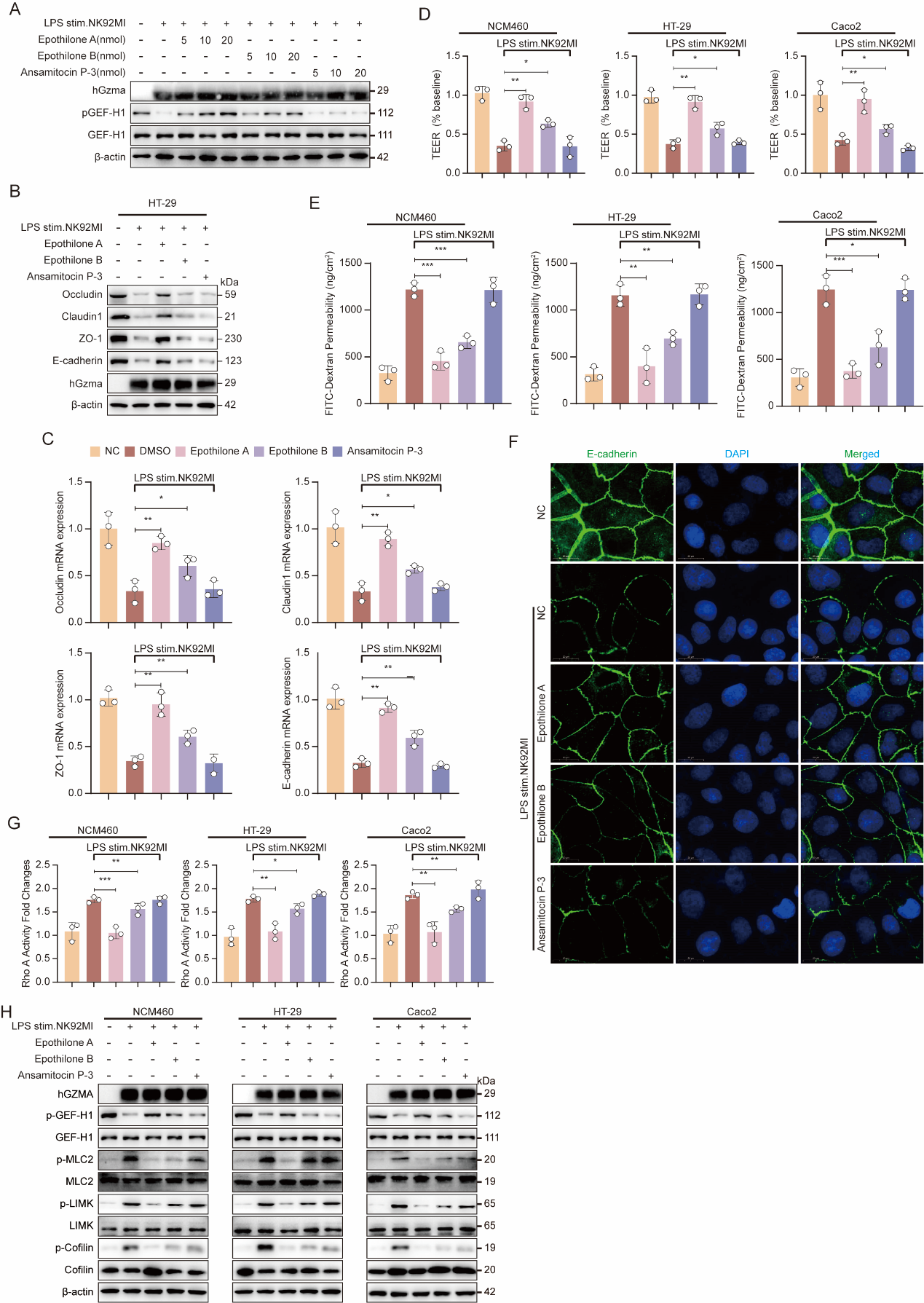

Supplement: Supplementary file 1 — Supporting Information [file CTM2-16-e70651-s001.docx]
